# Assessment of the Environmental Impact of Uranium Mining Sites: A Case Study of a Uranium Deposit in Southern Kazakhstan

**DOI:** 10.3390/toxics14080665

**Published:** 2026-07-27

**Authors:** Marina Krasnopyorova, Igor Gorlachev, Pavel Kharkin, Olga Milts, Sergey Lukashenko, Mariya Severinenko, Diana Akhmetzhanova, Amangul Bold, Valentina Slyadneva

**Affiliations:** 1 Institute of Nuclear Physics ARKAE, Ibragimov 1, Almaty 050032, Kazakhstan; 2 All-Russian Research Institute of Radiology and Agroecology of the National Research Center Kurchatov Institute, Russia, Obninsk 123098, Russia; 3 Faculty of Geography and Environmental Sciences, Al-Farabi Kazakh National University, Almaty 050040, Kazakhstan

**Keywords:** uranium mining, trace elements, radionuclides, ecological risk, lifetime cancer risk, soil analysis

## Abstract

To assess the environmental impact of uranium mining operations in southern Kazakhstan, the elemental and radionuclide composition of soil samples collected from settlements in the Kyzylorda Region was investigated. The analysis was carried out using X-ray fluorescence (XRF) and gamma-ray spectrometry. Mean concentrations and variation ranges were determined for 28 chemical elements, including uranium, lead, antimony, and gamma-emitting radionuclides such as ^137^Cs, ^40^K, ^232^Th, ^238^U, ^226^Ra, ^210^Pb, and ^241^Am. The analysis of the specific activities of the artificial radionuclides ^137^Cs and ^241^Am was carried out in order to assess the influence of the Semipalatinsk Test Site on the soils of the study area. Based on the obtained data, heavy metal pollution indices, ecological risk indices, and radiological parameters were calculated to evaluate the potential environmental and human health impacts. Most elements were present at levels below average crustal abundances, suggesting limited anthropogenic influence. Slight exceedances for uranium, lead, and antimony are likely associated with regional geochemical features. Radiological assessment indicated that the radiation environment remains within internationally accepted limits. The lifetime cancer risk values for exposure of humans to natural radionuclides ^226^Ra, ^232^Th, ^40^K and ^137^Cs from soil at 1 m above ground level ranged from 0.19 × 10^−3^ to 0.30 × 10^−3^, with an average of 0.24 × 10^−3^. Nearly all sampling points remained below the risk threshold of 0.29 × 10^−3^, indicating minimal radiological hazard. The carcinogenic risk remained within the acceptable regulatory range for both adults (2.0 × 10^−5^) and children (4.4 × 10^−5^), whereas the non-carcinogenic hazard index for children (1.3) slightly exceeded the screening threshold of 1. This finding identifies children as the most sensitive receptor group under the conservative assumptions of the applied screening methodology. The study demonstrates the applicability of combined chemical and radiometric methods for comprehensive environmental assessments in uranium mining regions. The results are of interest both in terms of methodology and in understanding the local geochemical and radiological landscape.

## 1. Introduction

One of the major global challenges today is the minimization of environmental risks and damage associated with modern energy production technologies. The Republic of Kazakhstan ranks first in the world in uranium production and second in uranium reserves [1]. According to the World Nuclear Association, Kazakhstan produced 22,808 tonnes of uranium in 2019, accounting for 41.66% of global output, while its estimated uranium reserves amounted to 842,200 tonnes in 2017 (14% of the world’s total) [2]. Currently, nearly all uranium produced in Kazakhstan is extracted using insitu leaching (ISL), which is considered the most environmentally and economically efficient method. However, due to the geological characteristics of uranium-bearing formations and the scale of both natural and anthropogenic processes, uranium ore provinces are classified as areas of elevated environmental risk, primarily owing to the potential release of radioactive and toxic elements into the environment.

During the development of uranium deposits, the primary environmental contaminants are uranium isotopes and their decay products, which exhibit both high radiological and chemical toxicity. Radioisotopes from all three natural radioactive decay series—^238^U, ^232^Th, and ^235^U—can be released into the environment through waste materials; however, radiological activity is primarily associated with the ^238^U series. Lead and polonium isotopes, as decay products of the ^238^U, ^232^Th, and ^235^U series via radon gas, are commonly found in uranium mining areas. These include relatively long-lived isotopes such as ^210^Pb and ^210^Po.

It is well established [3] that both acute and chronic uranium intoxication exert a multitropic effect on various organs and physiological systems. Soluble and insoluble uranium compounds produce similar types of damage, differing mainly in the onset and severity of toxic effects. In the early stages of exposure, chemical toxicity predominates, while radiological effects become more significant during prolonged exposure. Chronic radiation syndrome may developin cases of long-term internal exposure to poorly soluble uranium compounds.

In countries with significant uranium reserves such as India [4], Italy [5], Portugal [6], Saudi Arabia [7], the United States [8], Germany [9], and Bulgaria [10], a substantial body of data has been accumulated confirming the adverse effects of uranium and its decay products on human health. The ability of plants to hyperaccumulate uranium and transuranic elements from contaminated irrigation water and soil is also well documented globally [11,12,13,14,15].

Uranium mining is further associated with the release of heavy metals such as cadmium, manganese, lead, zinc, and copper, contributing to environmental contamination of soils, water bodies, sediments, and other environmental media [16,17,18]. In studies of contamination near mining operations, the most extensively investigated metals include Cd, Pb, As, Cu, Hg, Mn, and Ni [19,20], while rare earth elements (REEs, from La to Lu) and their associated risks generally receive less attention [21,22]. Nonetheless, uranium mining sites are often characterized by acid drainage that leads to enhanced mobilization of lanthanides [23] and actinides [24].

Soil is a vital natural resource with dual functions. On the one hand, it serves as a fundamental ecological component of ecosystems and a material basis for human survival. On the other hand, it acts as a sink for various environmental contaminants. Pollutants generated during mining activities can enter the biosphere via uptake by plants and agricultural crops, thereby altering the microecological structure of soils. These contaminants degrade soil fertility and ultimately affect the health and stability of entire ecosystems [25,26,27,28,29].

In the context of uranium extraction, the territory of southern Kazakhstan is characterized by the highest uranium potential, hosting three uranium ore provinces with deposits of various geological and genetic types. The current uranium mineral resource base of the Republic and its future potential are primarily represented by sandstone-type infiltration deposits located within the Shu-Sarysu and Syr Darya uranium provinces, situated in the Turkestan and Kyzylorda regions, respectively. For this study, the Kyzylorda Region was selected as the target area, which hosts four uranium deposits—Northern and Southern Karamurun, Irkol, and Northern Kharasan [1], with total uranium reserves estimated at 84,900 tonnes.

Unlike the uranium deposits in the Turkestan Region, mining enterprises in the Kyzylorda Region are situated in close proximity to relatively large settlements, such as the town of Shieli (population: 27,609) and Zhanakorgan (population: ~32,700), as well as 12 smaller communities. This geographic configuration presents a heightened risk of environmental and ecological impact on surrounding areas and may influence public health. Furthermore, part of the regional territory overlaps with protected natural areas, adding to the environmental sensitivity of the region.

In 2020, environmental specialists from LLP (Limited Liability Partnership) Institute of High Technologies conducted studies of the saline and chemical composition of soils in the area located south of the settlement of Shieli, near the North Karamurun deposit. According to the results, soil alkalinity, measured by the pH of aqueous extracts, ranged from 8.4 to 8.7. Measurements of the ambient dose equivalent rate H*(10)—a radiation protection quantity defined as the dose equivalent at a depth of 10 mm in the ICRU sphere, used for monitoring external photon radiation—indicated gamma radiation levels between 0.09 μSv/h and 0.20 μSv/h, which correspond to the natural background for this region.

At the North Karamurun deposit, the average concentrations in soils and sediments within the sampling interval down to 1 m depth were: ^238^U ranging from 139 to 153 Bq/kg, and ^232^Th ranging from 75 to 87 Bq/kg. The content of heavy metals determined by semi-quantitative spectral analysis did not exceed the maximum allowable concentrations (MAC) with the exception of arsenic, whose maximum concentration reached 20 MAC, and vanadium at 1.33 MAC. Significant exceedances of Clarke values were identified on average for barium by a factor of 1.4, arsenic by 5.98, copper by 2.0, and zinc slightly above 1.0 MAC [30].

In 2007, at the request of the Ministry of Environmental Protection of the Republic of Kazakhstan, radiometric surveys (alpha, beta, and gamma measurements) were carried out by the environmental consultancy “Ecoservis S” in the settlement of Shieli. No excess levels of radioactivity were detected: the maximum alpha particle count was 0.4 particles/cm^2^·min, beta particles—1.76 particles/cm^2^·min, and gamma background—0.17 μSv/h [31].

In 2008, the territories of eight settlements in the Shieli district were examined. The primary criterion for selecting the sampling locations was their proximity to uranium production facilities and the transportation routes of uranium-bearing solutions. Walkover gamma surveys indicated that background radiation levels generally ranged from 0.15 to 0.18 μSv/h. Elevated radiation was detected in the village of Shieli, where pipe fragments used by residents for fencing showed readings up to 24 μSv/h, and a coal ash pile near a residential building showed up to 0.67 μSv/h. In the village of Tartogai, even higher gamma radiation was recorded—up to 1.30 μSv/h—associated with a coal ash dump near a boiler facility.

In 2008, an assessment of heavy metal contamination in soils was also conducted in Shieli and Tartogai. The analysis revealed six sampling points with lead, copper, and zinc concentrations exceeding background levels by a factor of 3 to 4. The highest concentrations of lead (210 mg/kg) and zinc (220 mg/kg) were observed in Shieli, particularly near the asphalt plant and the storage tank area [32].

The studies described above highlight the significant role of anthropogenic factors in the contamination of soils in the investigated region. However, the scope and design of these studies were limited and lacked a systematic approach, and the resulting data were fragmentary, without attempting to characterise the overall situation across the entire region or to assess the potential impact of radionuclide and elemental contamination of soils on public health, which may be important for reducing risk perception and anxiety among populations living in close proximity to uranium mining facilities.

To identify possible elemental and radionuclide anomalies in the Syr Darya uranium province, we analyzed the elemental and radionuclide composition of 66 soil samples collected from 31 sites across the Kyzylorda Region. Based on the obtained data, we calculated heavy metal pollution indices, ecological risk indices associated with heavy metal contamination, and radiological parameters assessing the impact of naturally occurring gamma-emitting radionuclides on the environment and human health.

## 2. Study Area

**Figure 1:**
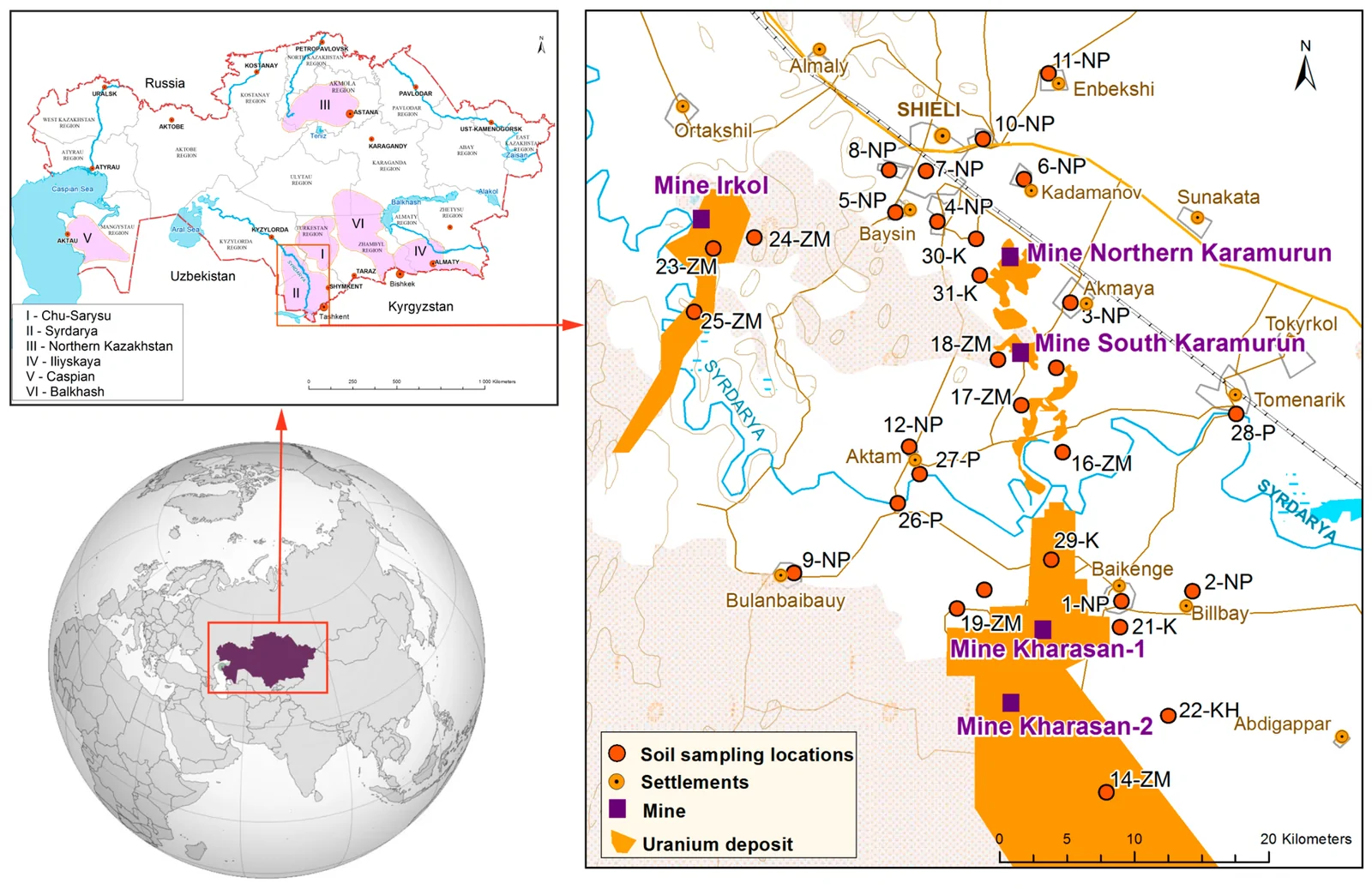
Location of the study area with designated sampling sites.

The Syr Darya uranium province is located in southern Kazakhstan, within the Kyzylorda Region, and covers an area of approximately 8000 km^2^. A map of the study area is shown in Figure 1. The province includes four uranium deposits: Northern Karamurun, Southern Karamurun, Irkol, and Kharasan. It is situated in the lower reaches of the Syr Darya River, near the southwestern foothills (Karamurun and Chaulinchi mountains) of the Greater Karatau Range.

From an orographic perspective, the ore field constitutes an extensive piedmont plain characterized by accumulative (depositional) landforms, adjacent to the Greater Karatau mountain range. The Syr Darya River crosses the area from the southeast to the northwest. The surface of the plain is predominantly composed of loamy and sandy soils, while loess-like sediments prevail in the floodplain of the Syr Darya. The most elevated zones are formed by sandy soils and are characterized by dune formations. In the Greater Karatau Mountains, the soils are rocky and stony. Absolute surface elevations within the ore field range from +170 to +175 m in the southeast to +150 to +160 m in the northwest.

The climate of the study area is sharply continental, characterized by large seasonal and diurnal temperature fluctuations and low annual precipitation—approximately 120 mm on the plains and up to 200 mm in the mountainous areas. Winters (December to February) are mild and have little snowfall. Daytime air temperatures typically range from −30°C to −8°C, while nighttime temperatures fall to −12°C to −18°C, with minimum recorded values reaching −36°C. Soil freezing can occur to depths of up to 1 m. Summers (May to September) are hot and dry, with clear, sunny weather. Daytime temperatures range from +22°C to +32°C, occasionally reaching as high as +42°C, and nighttime temperatures are generally between +12°C and +17°C. The area experiences persistent winds, predominantly from the northeast, with an average annual wind speed of 2.2–2.3 m/s [33].

The region is situated within a seismic zone of intensity VI on the Richter scale. The local economy is primarily based on irrigated agriculture and livestock farming. A significant portion of the left-bank floodplain has been developed for agricultural use, and a well-established network of irrigation canals is present.

The population of the Shieli and Zhanakorgan districts, both part of the Kyzylorda Region, is approximately 110,000 people, with the majority residing within the boundaries of the Syr Darya uranium province.

## 3. Materials and Methods

### 3.1. Sampling

When planning the sampling locations, priority was given to settlements, winter and summer pastures situated near mining lease areas, within potential impact zones, and along transportation routes used for the transfer of low-level radioactive waste to disposal sites, as well as to water sources used by the local population for domestic and drinking purposes. Secondary priority was given to the Syr Darya River, which serves as a recharge source for groundwater in the study area, along with the system of irrigation and drainage canals.

As part of the research, soil sampling sites were selected in the vicinity of the uranium mining operations at Northern and Southern Karamurun, Irkol, Kharasan-1, and Kharasan-2.

To ensure the representativeness of the study, as a rule, three sampling sites were selected in different parts of each settlement (in Shieli village—the largest settlement in the region—five such sites were selected). For each winter pasture (zimovka) and peasant farm, due to their small size, one to two sampling sites were selected. The riverbank soils of the Syr Darya River were sampled at six locations, and three sampling points corresponded to irrigation canals. In this way, we sought to cover the areas of local population settlement as fully as possible, so as not to miss potential elemental or radionuclide anomalies in the vicinity of inhabited areas. As shown in Figure 1, some sampling sites (14ZM, 29K, 31K, 25ZM, 23ZM) were located within or near the ore field of the uranium deposit lying at a depth of 350–700 m. We did not take samples near the injection and extraction wells of insitu leaching due to the restricted access to these areas. The number of sampling points could have been larger, which would have increased the representativeness of the sampling; the selected number—66—was determined by the capabilities of the analytical methods employed.

The sampling locations are shown in Figure 1, while the coordinates of the sites are provided in Supplementary Table S1 in the Supplementary Materials. A total of 31 sampling sites were selected, yielding 66 soil samples. Soil sampling was carried out using the envelope method from the surface layer (0–5 cm) over an area of 100 cm^2^, employing a sampler with dimensions of 10 × 10 × 5 cm in accordance with GOST 17.4.3.01-2017 [34]. Typically, 3 to 4 individual soil samples were collected at each sampling point and then combined into a composite sample to obtain the required amount of material. The total weight of each composite sample was no less than 1.0 kg. Samples were placed in fabric bags for geological sampling (30 × 40 cm) made of 100% cotton (plain weave) with a density of 210–230 g/m^2^, and labeled using a permanent marker on the outer surface. At each sampling location, geographic coordinates were recorded (GPS control), and photo documentation of the sampling process was performed.

In the analytical laboratory, soil samples were dried (drying conditions: +105°C until constant weight), sieved, and foreign inclusions such as stones, bones, etc., were removed. The samples were then ground using a disc mill to a particle size of 200 mesh.

### 3.2. Analytical Techniques

#### 3.2.1. X-Ray Fluorescent Analysis

For X-ray fluorescence (XRF) analysis, sample aliquots weighing no less than 15 g were taken from the pretreated material. The aliquots were transferred into sample cups with a diameter of 37 mm and a height of 10 mm, and placed directly above the detector for spectral measurements.

XRF analysis was performed using an ORTEC SLP10180 Si(Li) detector (ORTEC (AMETEK / Advanced Measurement Technology), Oak Ridge, TN, USA) with an active area of 80 mm^2^, an effective thickness of 5.0 mm, and an energy resolution of 175 eV at the 5.9 keV line (Kα of Mn), equipped with a 25 μm-thick beryllium entrance window. In our laboratory setup, radioactive isotopes ^109^Cd and ^241^Am are used as excitation sources. The use of different isotopes allows for the quantitative analysis of different groups of elements: from K to Mo, including W, Pb, Bi, Th, and U with ^109^Cd, and from Ag to Eu with ^241^Am.

To determine the count rates of analytical lines, we used the AnalX software for X-ray spectrum processing, developed at our institute [35,36]. The program is based on a least-squares fitting algorithm that models the X-ray spectrum using selected analytical functions representing the distribution of X-ray radiation. The software was developed as a Microsoft Windows application in the Delphi programming environment (©Inprise Corp., Borland Way, CA, USA). The linearization method involves expanding the analytical function into its first-order terms, with the calculation of correction coefficients for the initial estimates of nonlinearly evaluated parameters.

The uncertainties of the obtained elemental analysis results and the detection limits were calculated in accordance with the recommendations described in document [37], which is adopted as a regulatory guideline in the Republic of Kazakhstan for the validation of quantitative analytical methods. For this purpose, both international (IAEA–SL-1, IAEA–SL-3, IAEA–Soil-7, IAEA-405, IAEA-155, IAEA-RGU-1, IAEA-RGTh-1) and national (Kazakhstan) reference materials were used, with a total of 25 standards. As a result, the relative uncertainties of the data (Table 5) and the detection limits were calculated. Table S2 presenting the detection limits is included in the Supplementary Materials.

The analytical results of the measured concentrations of chemical elements were presented in the form: C ± Δ, where C is the concentration of the determined element (µg/kg), and Δ is the calculated uncertainty of the measured concentration (µg/kg). According to Table S2, the best detection limits achieved by XRF analysis are for La (1.1 µg/g), Ce, Zr, Nb, and Ba (1.2 µg/g), while the poorest detection limits are observed for K (3000 µg/g) and Ca (2000 µg/g).

#### 3.2.2. Gamma Spectrometry

For instrumental gamma-spectrometric analysis, the samples were placed in cylindrical measurement containers (d = 64 mm, sample mass—200 g). Gamma radiation photons were registered using a gamma spectrometer by CANBERRA, which includes a broad-energy germanium semiconductor detector (BeGe) (energy detection range: 3 keV to 3 MeV; energy resolution: 1.8 keV at the 1332 keV gamma line of ^60^Co), and a DSA-1000 multichannel digital signal analyzer.

Energy calibration was performed using a set of reference gamma sources: ^241^Am—59.5 keV, ^137^Cs—661.6 keV, ^60^Co—1173.2 and 1332.5 keV. Efficiency calibration of the detector was carried out using standard reference materials: IAEA-RGU-1, IAEA-RGTh-1, IAEA-300, IAEA-375, and IAEA-444.

Spectrum acquisition and analysis were conducted using specialized software Genie-2000 (version 3.4.1) in accordance with the measurement procedure registered under No. KZ 07.00.03126-2015. The measurement time for each sample in the experiments was 12 h.

The minimum detectable activity (MDA) levels for the analyzed radionuclides were as follows: ^241^Am—0.2 Bq/kg; ^137^Cs—0.3 Bq/kg; ^40^K—12 Bq/kg; ^232^Th—1.0 Bq/kg; ^238^U—2.0 Bq/kg; ^226^Ra—0.5 Bq/kg;^210^Pb—2.0 Bq/kg.

According to the method developed for measuring the activity concentrations of radionuclides in soil samples, the relative uncertainties of single measurements are less than 2% for ^40^K, 4.5% for ^232^Th, 5% for ^238^U, 2% for ^226^Ra, and 5% for ^210^Pb.

### 3.3. Quality Control and Assurance

Quality control of accuracy and precision in XRF analysis was carried out through the measurement of duplicate samples and the analysis of a certified reference material for trace elements in soil (Trace Elements in Soil, IAEA Soil-7). The final result of the analysis was taken as the average value of the duplicates. The results for the certified reference material were within the 95% confidence interval specified in the certificate.

To monitor the repeatability of elemental analysis, duplicate samples were measured at a ratio of 1 duplicate per 10 soil samples. Statistical processing of quality control data was performed using Microsoft Office Excel software.

The expanded uncertainty of activity measurements in gamma spectrometric analysis was calculated using the following formula [38]:

(1) $$∆A=\;2\sqrt{\sigma^{2}\left(\tilde{A}\right)\;+\Theta_{\Sigma }^{2}},$$

where σ(Ā)—is the Type A standard uncertainty of the activity measurement, determined by the uncertainty in the peak area $\sigma (S_{i})$: $\sigma \left(A_{i}\right)\;=\;\frac{\sigma (S_{i})}{\varepsilon (E_{i})\cdot t\cdot I_{i}}$;

$\Theta_{\Sigma }=\;\sqrt{\Theta_{\varepsilon }^{2}\;+\;\Theta_{l}^{2}\;+\;\Theta_{\rho }^{2}\;+\;\Theta_{m}^{2}}$—the quadratic sum of Type B standard uncertainties, which are calculated from relative uncertainties: $\Theta_{i}=\delta_{i}\cdot A,\;where$

$\delta_{\varepsilon }$—relative uncertainty of the detector’s registration efficiency;

$\delta_{l}$—relative uncertainty of the absolute gamma emission intensity values for the *i*-th line of the measured radionuclide;

$\delta_{\rho }$—relative uncertainty due to differences in self-absorption between the reference source and the measured sample;

$\delta_{m}$—relative uncertainty of sample mass measurement—considered only in the calculation of specific activity $A_{sp}=\frac{A}{m}$, m—sample mass.

To verify the accuracy of the obtained results, the specific activities of gamma-emitting radionuclides ^137^Cs, ^40^K, and ^241^Am were analyzed in standard reference materials of ore (IAEA-RGU-1, IAEA-RGTh-1) and soil (IAEA-375, IAEA-444, IAEA-Soil-6). The results obtained, presented in Table 1, are consistent with the certified values within the combined uncertainties of both the certified and measured activities. An exception was observed for ^241^Am in the IAEA-375 sample, where the detection limit exceeded the certified specific activity.

To monitor repeatability, control samples were measured at a ratio of 1:30.

### 3.4. Assessment of Heavy Metal Contamination

To assess the level of soil contamination by chemical elements, the following indices were calculated: single pollution indices—geoaccumulation index (I_geo_), contamination factor ($C_{i}^{f}$C); integrated (total) contamination indices—degree of contamination (C_deg_).

### 3.5. GeoaccumulationIndex

The geoaccumulation index (I_geo_) is used to assess contamination by comparing measured concentrations of chemical elements with their background levels. Originally developed for the evaluationof pollution in bottom sediments [39], it can also be applied to soil contamination studies. I_geo_ is calculated using the following equation:

(2) $$I_{geo}={log}_{2}\frac{C_{n}}{1.5B_{n}}$$

where C_n_ is the measured concentration of element *n* in the soil sample, and B_n_ is the geochemical background value of element *n*. The constant 1.5 is used to account for natural fluctuations in metal concentrations in the environment and to detect minor anthropogenic influences [39,40,41,42].

The geoaccumulation index includes seven contamination classes (Table 2):

### 3.6. Contamination Factor

To assess the degree of soil contamination, we also used the contamination factor ($C_{i}^{f}$C). The contamination factor is defined as the ratio of the mean concentration of an element in soil (based on no fewer than five samples) to its concentration in uncontaminated soil. Element concentrations in the Earth’s crust are commonly used as pre-industrial levels and are considered reference values for evaluating soil contamination by heavy metals [43,44].

(3) $$C_{f}^{i}=\frac{C_{0-1}^{i}}{C_{n}^{i}}$$

where $C_{0-1}^{i}$—the average concentration of the element from at least five sampling locations $C_{n}^{i}$—the pre-industrial concentration of a given element. Hakanson [43] defined four categories $C_{f}^{i}$:
$C_{f}^{i}$ < 1low contamination factor indicating low contamination1 ≤ $C_{f}^{i}$ < 3moderate contamination factor3 ≤ $C_{f}^{i}$ < 6Considerable contamination factor6 ≤ $C_{f}^{i}$Very high contamination factor

$C_{f}^{i}$—this is a single-element index. The sum of contamination factors for all investigated elements represents the degree of contamination (C_deg_) of the environment and is classified into four categories [43,44]:
C_deg_ < 8low degree of contamination8 ≤ C_deg_ < 16moderate degree of contamination16 ≤ C_deg_ < 32considerable degree of contamination32 ≤ C_deg_very high degree of contamination

### 3.7. Carcinogenic and Non-Carcinogenic Risks

Health risk assessment related to soil exposure is typically based on the evaluation of toxic effects from both non-carcinogenic and carcinogenic factors through three exposure pathways: ingestion, dermal contact, and inhalation [45,46]. Non-carcinogenic risk is represented by the hazard index (HI), which is calculated as the ratio of the chronic daily intake (CDI) of element *i* to the reference dose (RfD) for the corresponding exposure pathway.

Carcinogenic risk is represented by the total carcinogenic risk (CR), calculated as the product of the chronic daily intake (CDI) of element *i* and the slope factor (SF) [46,47,48].

The formulas are as follows:

Non-carcinogenic risk:

(4) $$HI=\sum {HQ}_{i,}$$

(5) $${HQ}_{i}=\frac{{CDI}_{i,ing/dermal/inh}}{RfD_{i,ing/dermal/inh}}$$

### 3.8. Carcinogenic Risk Assessment Methodology

(6) CR=∑CRi

(7) $${CR}_{i}={CDI}_{i,ing/dermal/inh\;}\times \;{SF}_{i,ing/dermal/inh\;}$$

where

HI—total non-carcinogenic hazard index;
HQ_i_—hazard quotient (HQ) of the *i*-th element;
CR_i_—carcinogenic risk index of the *i*-th element;
CDI_i_, ing/dermal/inh—chronic daily intake of the *i*-th element via ingestion, dermal contact, or inhalation (mg/(kg·day));
RfD_i_, ing/dermal/inh—reference dose of the *i*-th element via ingestion, dermal contact, or inhalation (mg/(kg·day));
SF_i_, ing/dermal/inh—slope factor of the *i*-th element via ingestion, dermal contact, or inhalation (mg/(kg·day))^−1^;

The subscripts ing, dermal, and inh indicate the exposure pathways: ingestion, dermal contact, and inhalation, respectively.

(8) $${CDI}_{i,ing}=\frac{Ci\times IngR\times EF\times ED}{BW\times AT}\times CF$$

(9) $${CDI}_{i,dermal}=\frac{Ci\times SA\times AF\times ABS\times EF\times ED}{BW\times AT}\times CF$$

(10) $${CDI}_{i,inh}=\frac{Ci\times InhR\times EF\times ED}{PEF\times BW\times AT}$$

The main parameters of the risk assessment model for non-carcinogenic and carcinogenic effects are presented in Table 3. The assessment was performed separately for adult and child population groups, taking into account age-specific differences in exposure parameters.

For the assessment of non-carcinogenic and carcinogenic risks, only elements with established reference doses (RfDs) and slope factors (SFs) according to international guidelines and scientific studies were considered (Table 4).

The threshold value for the non-carcinogenic risk level (HI) is 1 [53,54]. Accordingly, the individual risk of the *i*-th element must meet the criterion HQ < 1. To interpret carcinogenic risk (CR) values, the following classification was applied: CR < 1 × 10^−6^—no risk; 1 × 10^−6^ < CR < 1 × 10^−4^—tolerable risk; CR > 1 × 10^−4^—high risk [54,55]. A CR level below 1 × 10^−6^ indicates a probability of cancer occurrence of less than one case per one million people. Calculations and result visualizations were performed using Microsoft Office Excel.

### 3.9. Sensitivity Analysis Methodology

To assess the sensitivity of the deterministic health risk model to variations in the principal exposure parameters, a one-at-a-time (OAT) sensitivity analysis was performed [56,57]. The principal exposure parameters adopted from the US EPA methodology, namely contaminant concentration in soil (Ci), ingestion rate (IngR), body weight (BW), and exposure duration (ED), were individually varied by ±20%, while all other model parameters remained unchanged. A ±20% variation was selected to represent a moderate and plausible deviation from the baseline exposure assumptions, allowing the relative influence of each parameter on the estimated health risks to be evaluated without introducing unrealistic exposure scenarios. Model sensitivity was quantified as the percentage change in the Hazard Index (HI) and Carcinogenic Risk (CR) relative to the initial data estimates.

### 3.10. Evaluation of Radiological Parameters

#### Annual Effective Dose

The annual effective dose was calculated to estimate the biological effect per unit of absorbed dose, which depends on the type of radiation and the organs exposed. For members of the public, an annual effective dose limit of 1 mSv is recommended by the International Commission on Radiological Protection (ICRP) as part of the system of radiological protection introduced in ICRP Publication 60 and further refined in the 2007 Recommendations [58,59].

The dose is calculated using the following equation:

(11) $$E\left(\frac{mSv}{y}\right)=D\left(\frac{nGy}{h}\right)*\;8760\left(\frac{h}{y}\right)*\;0.2\;*\;0.7(\frac{Sv}{Gy})*\;10^{-6}$$

where D—is the absorbed dose rate in air (nGy/h), 8760 is the number of hours in a year, 0.2 is the outdoor occupancy factor, and 0.7 Sv/Gy is the conversion coefficient from absorbed gamma dose in air to effective dose for the whole body, as adopted by the ICRP. This calculation assumes that people spend 20% of their time outdoors and 80% indoors.

### 3.11. Excess Life Time Cancer Risk

Prolonged exposure to radiation is believed to carry an increased risk of cancer. According to the National Cancer Institute [60], the lifetime risk of developing cancer is 44% for American men and 38% for women. The “Excess Lifetime Cancer Risk” (ELCR) refers to the additional risk of developing cancer that may arise from long-term exposure to radiation. In accordance with the provisions discussed in [61,62], the excess lifetime cancer risk is calculated based on the estimated annual effective dose values using the following equation:

(12) ELCR=E ∗ DL ∗ RF

where E—is the annual effective dose (mSv/y), DL—is the expected duration of life, approximately 70 years, and RF—0.05 Sv^−1^ was adopted, consistent with the nominal risk coefficients for stochastic effects recommended by the ICRP in Publication 60 and essentially maintained, with updated age- and tissue-specific detail, in the 2007 Recommendations [58,59].

### 3.12. Statistical Analysis

Inter-element relationships can provide valuable insights into the sources and pathways of heavy metals and radionuclides in soil [63]. To identify such relationships, we conducted a correlation analysis using the Statistica software package (version 10.4). The Statistica software allows for various types of data analysis, including descriptive statistics, regression analysis, factor analysis, and cluster analysis. Descriptive statistics provide an overall view of the dataset, regression analysis helps to identify dependencies between variables, and factor analysis is used to uncover latent factors influencing the data. Cluster analysis is a multivariate statistical technique for grouping a broad range of complex data into several clusters with similar characteristics or for identifying common sources of pollution [64]. In this study, cluster analysis was applied to interpret complex relationships between heavy metals and radionuclides and to identify their potential sources.

### 3.13. Spatial Distribution Mapping

Spatial-distribution maps of element concentrations and radionuclide activities were produced in ArcGIS 10.4 (Esri, Redlands, CA, USA) using Natural Neighbor interpolation [65]. For each query point the algorithm selects the closest subset of input samples and weights them by proportionate area (Sibson’s method). It is a local interpolator, and the estimated values are constrained to the range of the input samples, so that no artificial maxima or minima are introduced. This method was selected because it requires no assumption about the underlying spatial model and performs robustly for the irregularly distributed sampling network used in this study. As a purely local, range-bounded interpolator, it is also well suited to confirming the absence of spatial anomalies rather than imposing apparent structure on a near-uniform field. Where several sampling sites fell within a single settlement, the values were averaged prior to interpolation. Class boundaries on the map legends were defined using equal intervals.

## 4. Results and Discussion

### 4.1. Heavy Metal Content in Soil Samples

The concentrations of heavy metals in the soils of the studied region are presented in Table 5. As shown in Table 5, the average concentrations of elements across the region are generally consistent and, for some elements, even lower than their concentrations at the background site. No anomalous variations in concentrations were observed. The greatest deviation of maximum concentration from the background level was recorded for strontium, with the maximum value exceeding the background concentration by a factor of 2.3.

Thus, based on Table 5, it can be concluded that there is no significant impact of the uranium deposit or uranium mining activities on the elemental composition of soils in areas located near the uranium mines. For uranium, the relative standard deviation is 14%, with a Max/Min ratio of 2.14, indicating a fairly stable distribution of uranium across the study area.

For most chemical elements, the average concentrations in the region are lower than their contents in the upper continental crust [66]. The mean uranium concentration exceeds that of the upper continental crust by a factor of 1.2, while the maximum exceeds it by a factor of 2.2. The highest exceedances of mean concentrations over those in the upper continental crust were observed for antimony (5.7 times) and lead (3.1 times), which may be considered a regional characteristic of the soil elemental composition.

**Figure 2:**
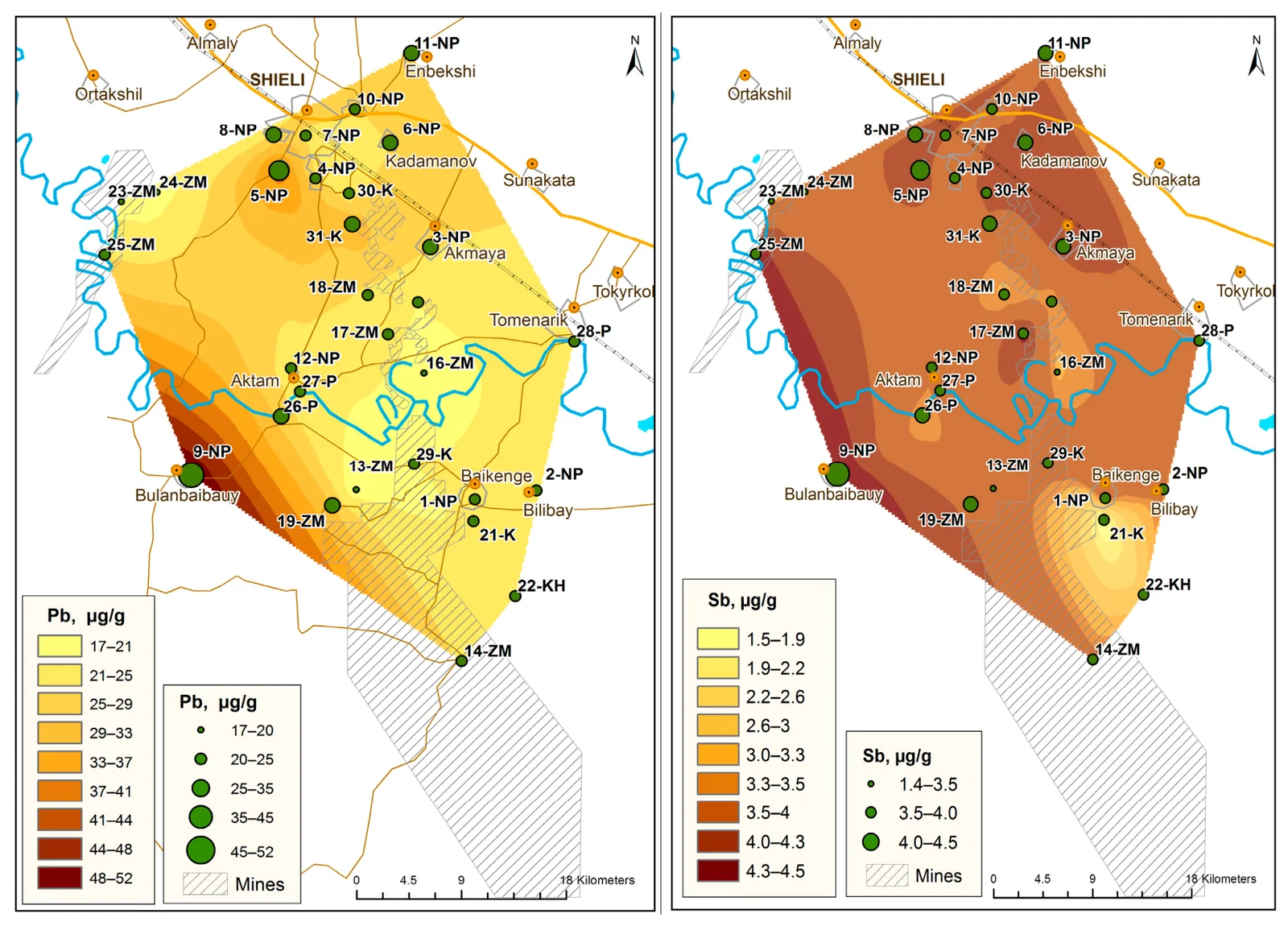
Spatial distribution of lead (**left**) and antimony (**right**) in soils of the study area, µg/g. Graduated symbols show settlement-averaged concentrations at sampling sites; the colored surface is the Natural Neighbor interpolation; hatching denotes the mine lease areas.

Figure 2 presents the spatial distribution of the mean concentrations of Pb and Sb for each sampling site, as these elements demonstrated the most significant enrichment relative to the upper continental crust among all analysed heavy metals. Where several sampling points were available within a given settlement, the concentration values were averaged. As shown in Figure 2, the highest concentrations of both elements occur at the same sites—9-NP (52 µg/g for Pb and 4.6 µg/g for Sb) and 5-NP (40 µg/g for Pb and 4.3 µg/g for Sb). However, when the region is considered as a whole, the correlation between lead and antimony is less pronounced, as shown in Table 13 and Figure 6: the Pearson correlation coefficient between Pb and Sb is r = 0.69, which does not allow an unambiguous conclusion about a common origin of these elements.

The interpolated surfaces for both elements are smooth and show no gradient oriented toward the ore field or the mine lease areas; the local maxima occur in the northwest of the study area, remote from the leaching operations. This spatial pattern is consistent with a local geochemical feature of the soil parent material rather than with a mining-related source.

Elements such as Mo, Sn, and Ba also show mean concentrations exceeding UCC reference values (Table 5); however, their exceedances are considerably less pronounced and are attributed primarily to natural geochemical background consistent with the lithological characteristics of the study area, rather than anthropogenic impact.

### 4.2. Radionuclide Activity Concentrations in Soil Samples

Summary statistics of the specific activities of gamma-emitting radionuclides in the soils of the studied region are presented in Table 6. The analysis of the specific activities of the artificial radionuclides ^137^Cs and ^241^Am was carried out in order to assess the influence of the Semipalatinsk Test Site on the soils of the study region.

At all sampling points, the activity of ^241^Am does not exceed the minimum detectable activity level (0.5 Bq/kg). The global fallout level in the upper 20 cm of soil ranges from 0.02 to 5.0 Bq/kg for ^241^Am and from 4 to 29 Bq/kg for ^137^Cs [67,68,69,70,71]. Thus, the concentrations of both radionuclides in the soils of the studied region do not exceed the upper limit of global fallout levels.

Among all the radionuclides studied, ^40^K exhibits the highest specific activityin the investigated area.

Table 7 presents the concentrations of natural radionuclides in soils from various countries around the world, along with the data obtained in our study for soils of southern Kazakhstan [72,73,74]. The results indicate that the soils of southern Kazakhstan do not exhibit anomalous concentrations of natural radionuclides, and the maximum values of specific activities do not exceed the upper limits of activity ranges reported for soils in other regions of the world.

**Figure 3:**
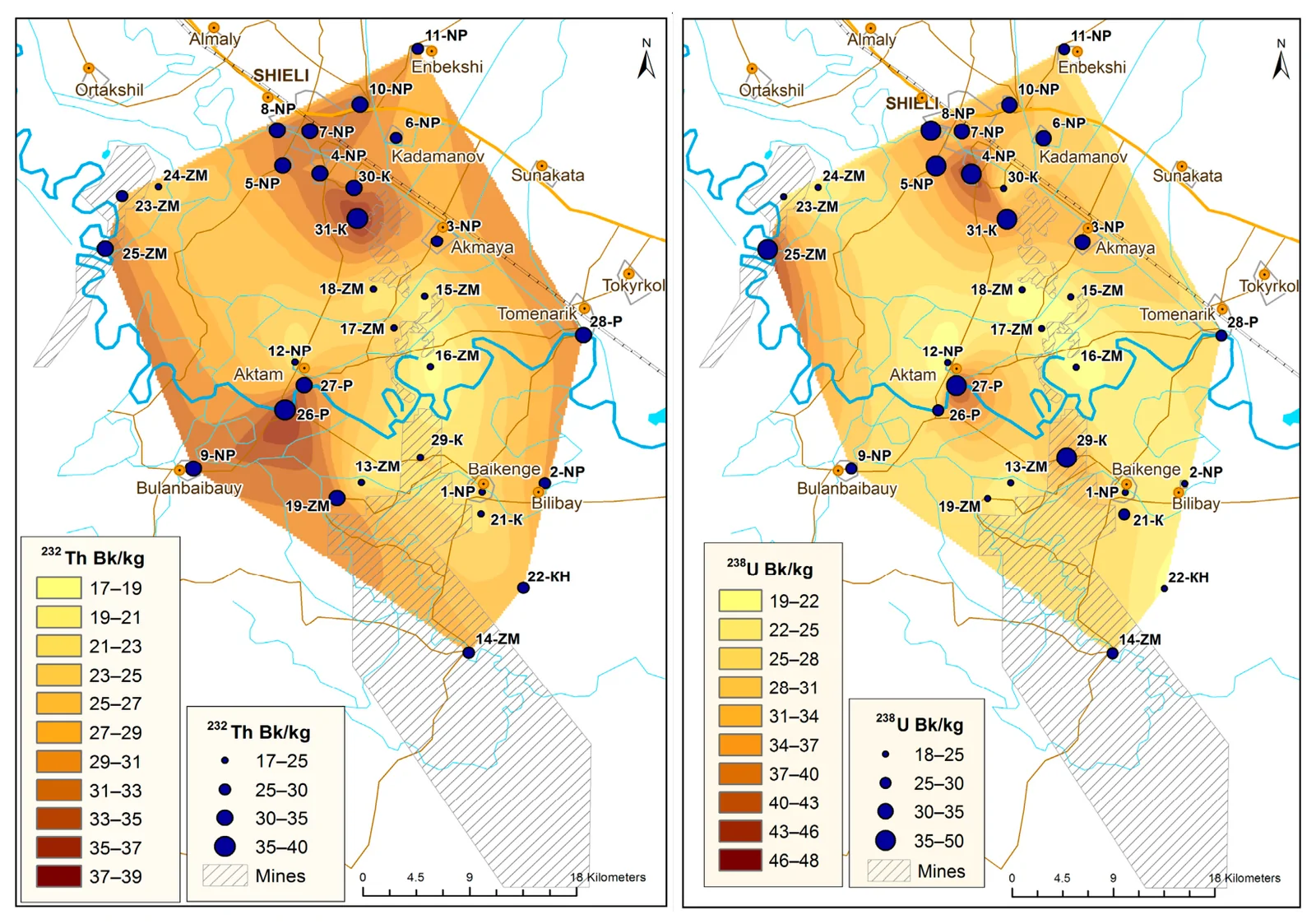
Spatial distribution of the specific activities of ^232^Th (**left**) and ^238^U (**right**) in soils of the study area, Bq/kg.

Figure 3 shows the specific activities of ^238^U, ^232^Th in the studied settlements of southern Kazakhstan. If multiple sampling points were available within a given settlement, the specific activity values were averaged.

As shown in Figure 3, the highest mean concentrations of ^238^U and ^232^Th occur at the same sites—27-NP (33 Bq/kg for ^232^Th and 48 Bq/kg for ^238^U), 31-K (40 Bq/kg for ^232^Th and 39 Bq/kg for ^238^U), and 4-NP (30 Bq/kg for ^232^Th and 50 Bq/kg for ^238^U). The correlation between ^238^U and ^232^Th is also fairly well pronounced, as shown in Table 13 and Figure 7: the Pearson correlation coefficient between ^238^U and ^232^Th is r = 0.61. In contrast, the Pearson correlation coefficient between elemental uranium and thorium (Table 13 and Figure 6) is r = −0.01, indicating a weak relationship between the elements.

The observed similarity in the spatial distribution of ^238^U and ^232^Th in the soils of the study area emphasises that the location of the uranium deposits has no influence, at the regional scale, on the radionuclide composition of the surface soil layer. The locally elevated concentrations of both ^238^U and ^232^Th most likely reflect the natural heterogeneity of the spatial distribution of radionuclides.

**Figure 4:**
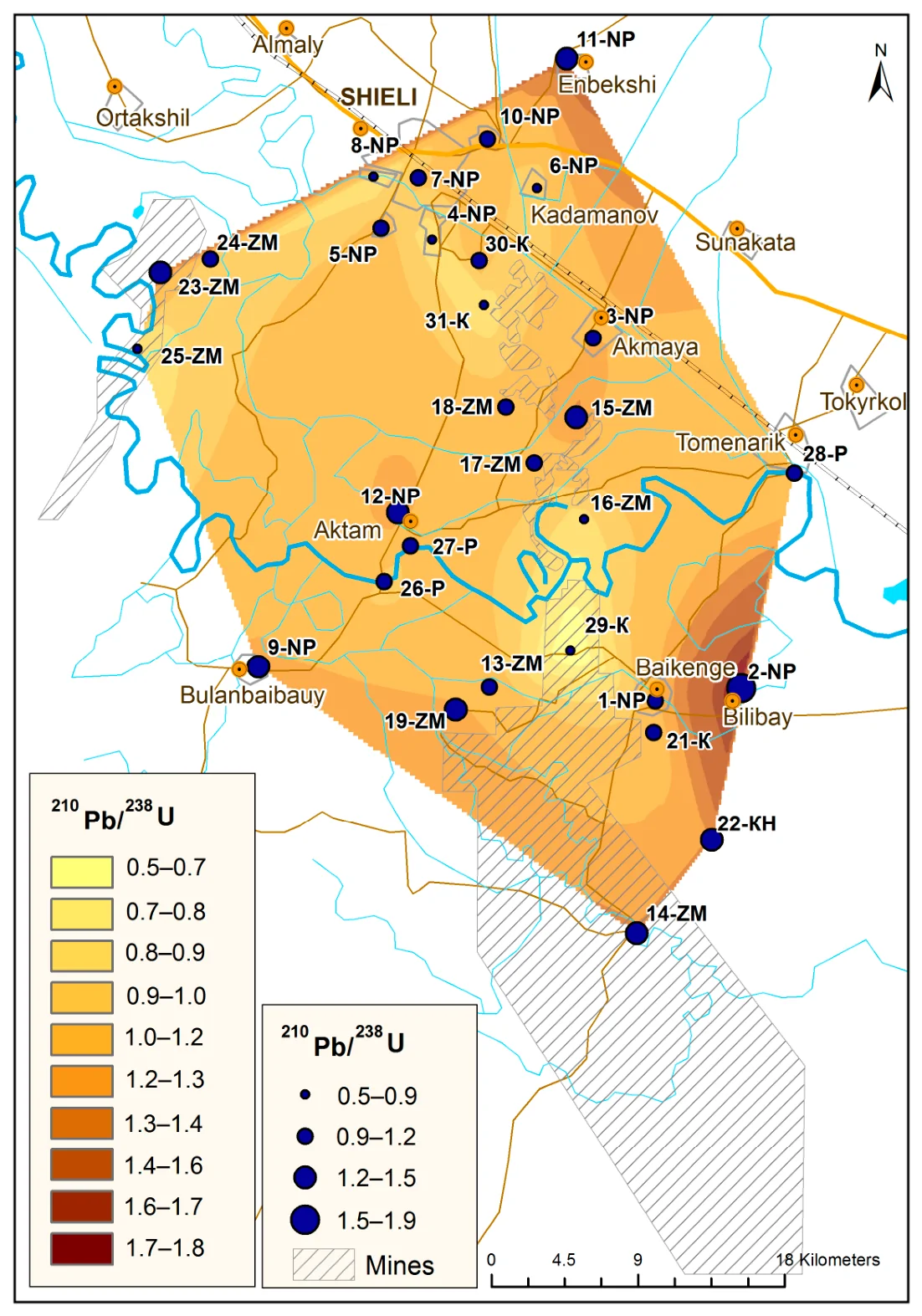
Spatial distribution of the ^210^Pb/^238^U activity ratio in soils of the study area.

Across the interpolated ^232^Th and ^238^U surfaces there is likewise no systematic increase in the vicinity of the mine lease areas, and the weakly elevated values are distributed irregularly over the province. The scattered pattern of the ^210^Pb/^238^U ratio (Figure 4), which exceeds unity at isolated sites with no spatial relationship to mining infrastructure, further supports a ^222^Rn-mediated surface-enrichment origin rather than a mining source. Taken together, the spatial distributions reinforce the conclusion that insitu leaching exerts no detectable regional-scale influence on the elemental or radionuclide composition of the surface soil.

Figure 4 presents the activity ratios of ^210^Pb to ^238^U in different settlements. The radioactive isotope ^210^Pb is a decay product of ^238^U. Therefore, under equilibrium conditions, the concentrations of these isotopes in soil should be approximately equal. The observed exceedance of ^210^Pb specific activity over that of ^238^U (up to 2 times in point 2NP), as shown in Figure 4, suggests the possible presence of mechanisms leading to the enrichment of surface soil layers with ^210^Pb. This phenomenon can be explained by the presence of the gaseous radionuclide ^222^Rn in the decay chain between ^238^U and ^210^Pb, which has a half-life of 3.82 days. During its lifetime, radon gas may migrate to the surface through cracks in the soil. After approximately four days, ^222^Rn decays to ^210^Pb, which settles in the surface soil layer, leading to its enrichment with ^210^Pb.

**Figure 5:**
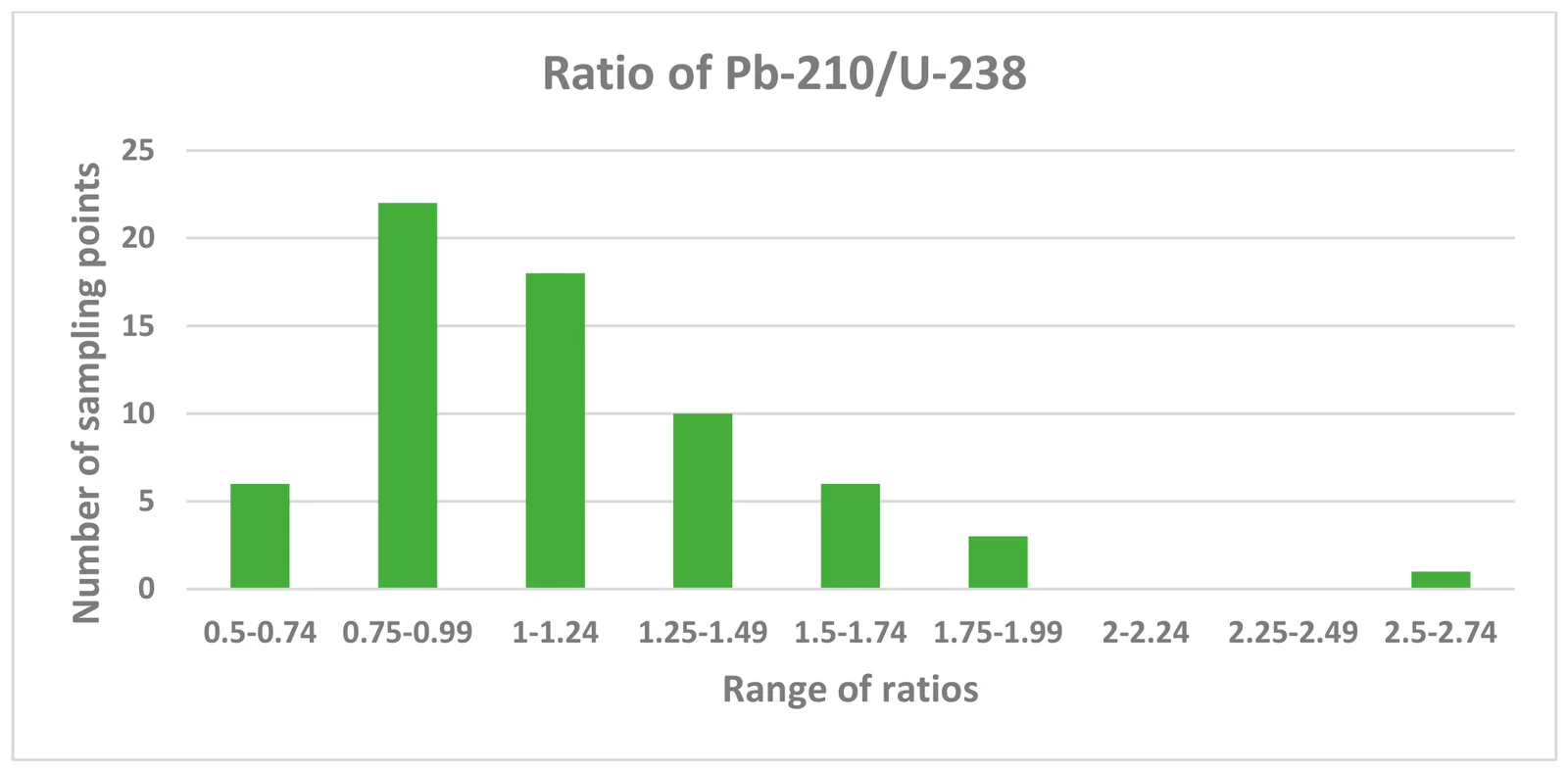
Frequency distribution of sampling points by the ^210^Pb/^238^U activity ratio.

This conclusion is further supported by Figure 5, which shows the distribution of sampling points by the range of the ^210^Pb/^238^U activity ratio. The deviation of the distribution from a Gaussian shape, with an extended tail in the range where ^210^Pb activity exceeds ^238^U activity, indicates that in some locations, mechanisms of ^210^Pb enrichment in soil are indeed at work.

### 4.3. Heavy Metals Contamination

#### 4.3.1. Geoaccumulation Index Calculation

To assess the degree of soil contamination at selected sampling points, the geoaccumulation index (Igeo) was calculated according to Equation (2). As background values, the concentrations of chemical elements from sampling point 9 (Figure 1 and Table 1), located at a relative distance from uranium mining areas, were used. For nearly all elements and sampling points, the condition Igeo ≤ 0 is satisfied, indicating that the soil is practically uncontaminated.

Exceptions include point 4 (Figure 1) for vanadium, point 30 for zirconium, and points 30 and 31 for thorium, where 0 ≤ Igeo< 1. In these cases, the soil quality can be characterized as ranging from uncontaminated to moderately contaminated.

At sampling points 25, 26, 27, and 28, Igeo ≤ 0 for all studied chemical elements. These sampling sites are located near the Syr Darya River. Therefore, it can be concluded that the influence of the river on the elemental composition of the adjacent soils is minimal.

#### 4.3.2. Contamination Factor Calculation

The degree of soil contamination in the studied region relative to pre-industrial levels of element concentrations is determined using the contamination factor, calculated according to Equation (2).

Table 8 presents the contamination factors for 12 chemical elements for which pre-industrial levels are defined.

As shown in Table 8, for almost all elements, the soil contamination level in the region falls into the category of “low contamination.” Exceptions are manganese and praseodymium, for which the contamination level is classified as moderate. Both elements exhibit consistently elevated concentrations at all sampling points compared to pre-industrial levels: the mean Mn concentration in the region’s soil is 457 µg/g with a standard deviation of 79 µg/g, while for Pr these values are 9.4 µg/g and 1.1 µg/g, respectively. This suggests that the relatively high concentrations of Mn and Pr are characteristic features of the soil in the studied region.

The calculated integral parameter—the degree of contamination (Cdeg), defined as the sum of individual contamination factors for all studied elements—is Cdeg = 10, which corresponds to a moderate level of soil contamination in the study area.

### 4.4. Assessment of Carcinogenic and Non-Carcinogenic Risks

When interpreting the results of risk assessments, particularly carcinogenic risks, it is important to consider methodological limitations. The models used (US EPA) are based on the linear no-threshold concept, which serves as a conservative tool for risk management under elevated exposure conditions. However, at contaminant concentrations comparable to the regional geochemical background, the calculated values of carcinogenic risk (CR) are predominantly stochastic in nature. This means that they represent a theoretical, probabilistic estimate of additional disease cases in a large population over a lifetime, rather than a prediction of inevitable disease development in a specific individual. Therefore, the presented numerical CR values should be interpreted as indicators of the relative contribution of different elements and exposure pathways, with their absolute magnitude considered in the context of acceptability for the population as a whole.

### 4.5. Non-Carcinogenic Risk

The potential health risks associated with soil contamination were evaluated for adult and child populations considering three primary exposure pathways: ingestion, dermal contact, and inhalation (Table 9).

Interpretation of the obtained HQ/HI values requires consideration of methodological context. The hazard quotient model is a conservative screening tool based on reference doses (RfD) that incorporate substantial uncertainty factors designed to protect sensitive population groups. Therefore, an HQ or HI value exceeding unity should be regarded as a screening indicator identifying the need for further evaluation rather than direct evidence of adverse health effects.

The total Hazard Index (HI) was 0.15 for adults and 1.3 for children. While the HI for adults indicates no concern, the value for children exceeds the threshold of 1. This result reflects the greater susceptibility of children due to higher soil ingestion rates and lower body weight, which increase the chronic daily intake per unit body mass.This result must be interpreted with caution, considering that: (a) the primary contributors (Fe, Cr, V) are naturally occurring elements with concentrations at the regional geogenic background level; and (b) the RfD values are inherently conservative. Thus, an HI > 1 in this context serves as a precautionary indicator identifying oral intake as the dominant pathway and children as a more sensitive group [50,75]. The exceedance of the Hazard Index above unity for children indicates a greater susceptibility of the child’s organism to external contamination factors, owing to the fact that the chronic daily intake (CDI) of contaminants is calculated per unit body weight (parameter BW in Equations 8, 9, and 10).

### 4.6. Carcinogenic Risk Results and Discussion

The assessment of carcinogenic risk, performed for Chromium (Cr) and Lead (Pb), employs a linear non-threshold model, a conservative tool for regulatory decision-making. It is crucial to note that at exposure levels comparable to the natural background, as in the present study, any potential carcinogenic effect would be stochastic (probabilistic) in nature. The calculated CR values represent a theoretical lifetime probability of additional cancer cases in a large population and should not be misinterpreted as a deterministic prediction of individual disease.

The results of the carcinogenic risk index (CR) calculation for adults and children are presented in Table 10.

The calculated total carcinogenic risks are 2.0 × 10^−5^ for adults and 4.4 × 10^−5^ for children. According to the US EPA classification, these values fall within the “acceptable” or “negligible” risk range (10^−6^–10^−4^), which is considered manageable for population-level regulation and is comparable to many commonplace risks. The risk for children is approximately twice that for adults, attributable to higher intake rates per body weight and specific behavioral factors.

### 4.7. Sensitivity Analysis Results and Discussion

OAT sensitivity analysis was performed to evaluate the influence of the principal exposure parameters on the deterministic health risk estimates (Supplementary Table S3). A ±20% variation in the selected exposure parameters resulted in changes of up to ±20% in both the Hazard Index (HI) and Carcinogenic Risk (CR) due to variations in contaminant concentration (C_i_), up to ±25% due to body weight (BW), and approximately ±17–19% due to ingestion rate (IngR). Variations in exposure duration (ED) affected only carcinogenic risk (±20%) and had no influence on the Hazard Index. Overall, contaminant concentration (C_i_) and body weight (BW) were identified as the most influential parameters. Despite these variations, the interpretation of the estimated risks remained unchanged: the CR remained within the acceptable regulatory range for both population groups, whereas the HI for children consistently remained above the screening threshold.

### 4.8. Radiological Parameters

Table 11 presents the mean, standard deviation, maximum, and minimum values of the investigated radiological parameters.

### 4.9. Calculation of Annual Effective Dose

The Annual Effective Dose resulting from the presence of radioactivity in the collected soil samples was calculated using Equation (11). The maximum Annual Effective Dose among the soil samples was 0.087 mSv/y, while the minimum was 0.053 mSv/y. The average value across all samples was 0.067 mSv/y. The recommended average annual dose for an individual, according to the International Commission on Radiological Protection (ICRP), is 1 mSv/y [58]. In this study, the calculated Annual Effective Dose for all sampling points did not exceed the ICRP recommended value.

At sampling points 25, 26, 27, and 28, located near the Syr Darya River, the dose ranged from 0.067 to 0.074 mSv/y, indicating no significant anthropogenic contamination of the region’s coastal soils by radionuclides from the Syr Darya River. Concerns about possible contamination of the floodplain soils of the Syr Darya River are driven by previous studies reporting the presence of toxic elements and radionuclides in Syr Darya River water samples [76,77].

The largest contribution to the annual effective dose is made by K-40, whose contribution to parameter D in Equation (11) ranges from 36% to 58%, while the smallest contribution is that of Cs-137, ranging from 0.009% to 0.35%.

### 4.10. Life Time Cancer Risk

The Excess Lifetime Cancer Risk (ELCR) in the studied area ranges from 0.19 × 10^−3^ to 0.30 × 10^−3^**,** with an average value of 0.24 × 10^−3^ (Table 11). For most sampling points, the ELCR does not exceed the established threshold value of 0.29 × 10^−3^ [78], above which a slight increase in cancer risk has been reported. Exceedances were observed at only two sampling points—ELCR = 0.30 × 10^−3^ at locations 7NP and 19 ZM (see Figure 1).

### 4.11. Comparison with Uranium Mining Areas Worldwide

To place the present findings in a global context, the radiological parameters were compared with published data from uranium mining regions using different extraction technologies (Table 12). The mean annual effective dose obtained here (0.067 mSv/y) is close to the world average outdoor value of 0.07 mSv/y (UNSCEAR) and substantially lower than values from conventional mining areas. At the Rössing open-pit mine (Namibia), annual effective doses in soils averaged 0.30 mSv/y and reached 1.60 mSv/y; in the underground mining districts of Singhbhum (India) they ranged from 0.11 to 0.14 mSv/y; and in a uranium mining area of South China radiological indices exceeded world averages by a factor of two to ten.

The mechanism behind these differences is instructive. At Jaduguda, the ELCR in surrounding soils (0.27 × 10^−3^) remained below the UNSCEAR threshold, whereas tailings and waste rock exceeded it (0.39 × 10^−3^ and 0.37 × 10^−3^), and monitoring at Rössing attributed elevated radionuclide levels in nearby soils to wind-blown fines from the tailings. Both tailings and waste rock are absent insitu leaching (ISL), where ore is dissolved underground and not brought to the surface.

Our ELCR values (mean 0.24 × 10^−3^, range 0.19–0.30 × 10^−3^) exceed the UNSCEAR threshold of 0.29 × 10^−3^ at only two sites. Systematic radiological assessments of surface soils in ISL areas remain scarce, as the literature is dominated by open-pit and underground mining and ISL research has focused mainly on groundwater. The present study thus addresses this gap and shows that, despite active ISL operations in the Syr Darya province, the radiological burden on surrounding soils remains typical of background territories. Soil contamination at ISL sites has nonetheless been reported elsewhere in association with surface infrastructure and solution spills, underlining the importance of the monitoring presented here.

### 4.12. Correlation Analysis

**Figure 6:**
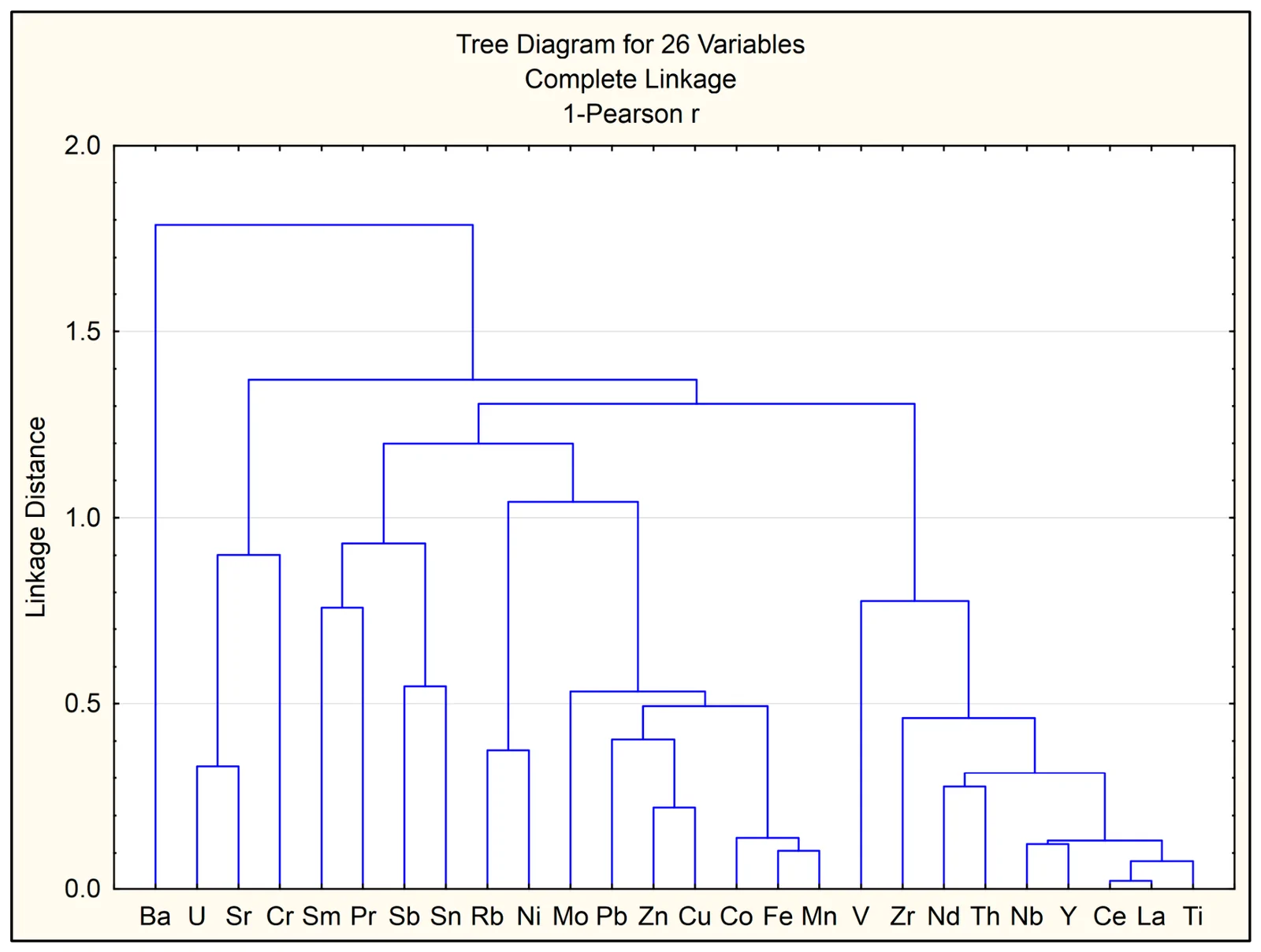
Cluster Analysis of Chemical Elements.

**Figure 7:**
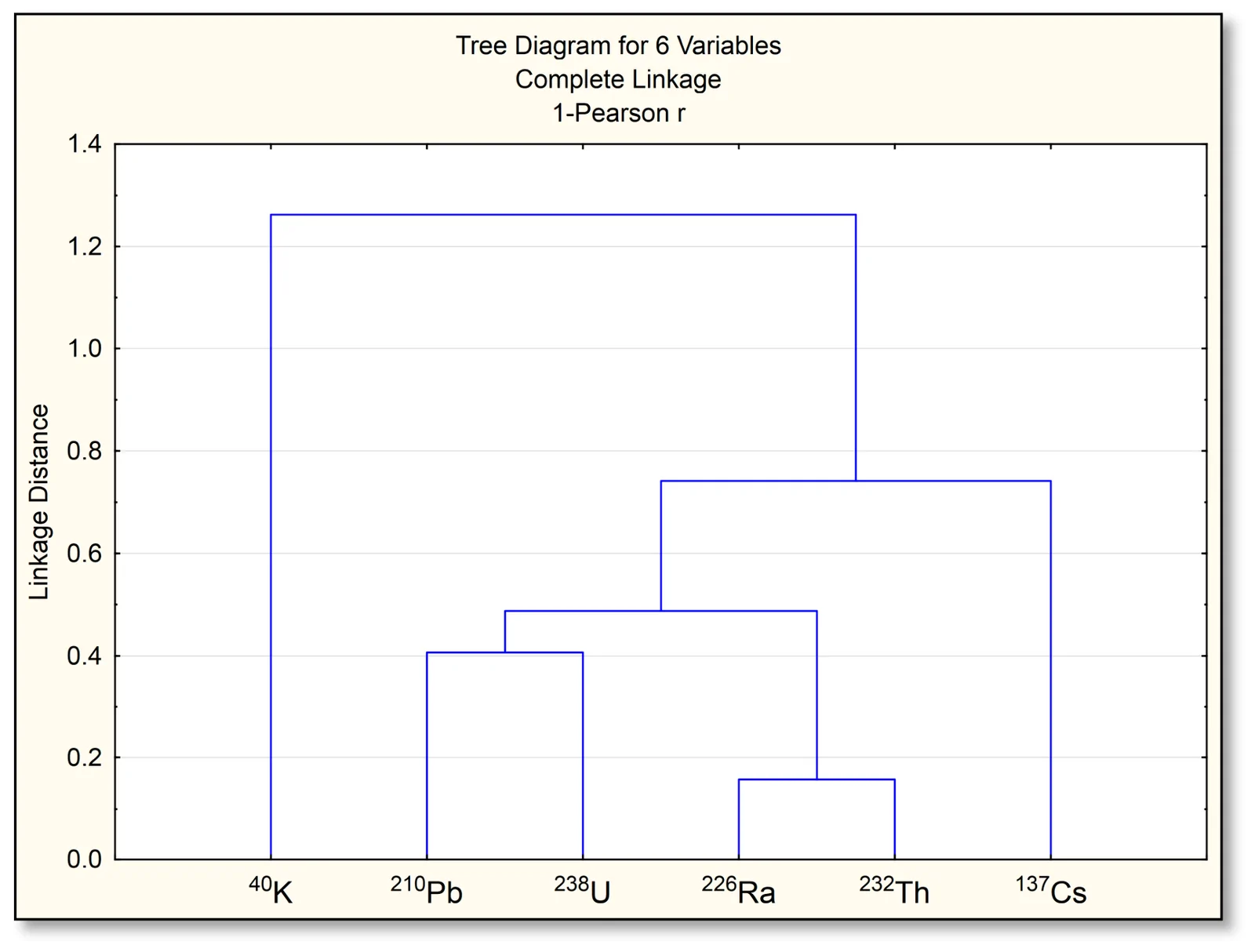
Cluster Analysis of Radionuclides.

To identify distinct groups of heavy metals and radionuclides of natural or anthropogenic origin, Pearson correlation analysis was applied, and the results are presented in Table 13 (for elements) and Table 14 (for radionuclides). Based on the data in Table 13 and Table 14, a cluster analysis of the chemical elements and gamma-emitting radionuclides was conducted, as shown in Figure 6 and Figure 7, respectively. The vertical axis of the figures represents the distance, calculated as 1–r; the smaller the distance between the investigated objects, the stronger the correlation between them.

Strong correlations were observed between the chemical elements: La-Ce (1 − r = 0.02), La-Ti (1 − r = 0.07), Ce-Ti (1 − r = 0.08), Nb-Y (1 − r = 0.11), Mn-Fe (1 − r = 0.11), Mn-Pb (1 − r = 0.19) and Pb-Fe (1 − r = 0.11). These strong correlations between certain heavy metals indicate similar sources of origin and geochemical behavior [82,83]. According to Table 13 and Figure 6, two main clusters of chemical elements were identified. The first includes La, Ce, Ti, Nb, Y, Th, Nd, and Zr. The strong positive correlations among these elements in soils suggest that they have similar contamination levels and likely share common sources of predominantly natural origin [84]. The second cluster includes Co, Fe, Mn, Cu, and Zn; the covariation of these elements is consistent with natural Fe-Mn (hydr)oxide scavenging rather than an anthropogenic contribution. Uranium does not show high correlation with other elements; the highest observed association is in the U-Sr pair (1 − r = 0.47).

Strong correlations were also found among radionuclides: ^226^Ra-^232^Th (1 − r = 0.16), ^238^U-^232^Th (1 − r = 0.39), and ^238^U-^210^Pb (1 − r = 0.41) (Table 14). ^210^Pb and ^226^Ra are decay products of the natural isotope ^238^U, which logically explains the correlation among these radionuclides. The strong correlation between ^238^U and ^232^Th is likely due to the presence of ^232^Th in uranium deposits in southern Kazakhstan.

Radionuclides ^137^Cs and ^241^Am are mainly products of nuclear fission of ^235^U and ^241^Pu, respectively, in nuclear reactors and during nuclear weapons use. Their presence in the Earth’s crust is due to global fallout. Consequently, ^137^Cs does not correlate with naturally occurring radionuclides, as shown in Table 14 and Figure 7: for the ^238^U-^137^Cs-pair, the correlation coefficient is 1 − r = 0.67.

^40^K, like ^238^U, is a naturally occurring isotope. It is believed that all existing ^40^Kon Earth formed shortly before the birth of the Solar System and the planet itself, approximately 4.54 billion years ago. Therefore, this isotope should be relatively evenly distributed throughout the Earth’s crust and is not genetically associated with uranium deposits. This is confirmed by Table 14 and Figure 7, which show a very low correlation between ^40^K and other studied radionuclides (correlation coefficient for ^40^K-^238^U is 1 − r = 1.26).

## 5. Conclusions

For most chemical elements, the average concentrations across the study area were lower than their average values in the upper continental crust. However, the mean uranium concentration exceeded the crustal background level by a factor of 1.2, and the maximum value was 2.2 times higher. The most significant exceedances were observed for antimony (5.7 times higher) and lead (3.1 times higher), which may reflect regional geochemical characteristics of the soil composition.

For almost all elements and sampling locations, the condition I_geo_ ≤ 0 is met, indicating that the soils are practically uncontaminated. Only two sites exhibit values in the range 0 ≤ Igeo < 1, suggesting an intermediate status between uncontaminated and moderately contaminated. In terms of the contamination factor (CF), the degree of soil contamination for nearly all elements falls into the “low contamination” category. Thus, it can be concluded that, in terms of elemental composition, the soils in southern Kazakhstan are generally in a favorable condition, and the presence of uranium deposits and mining operations in the region does not significantly affect soil quality.

The total non-carcinogenic risk index was 0.15 for adults and 1.3 for children, against a threshold value of 1. This indicates no significant health risk for the adult population, while a minor potential threat of chronic exposure to toxic elements may exist for children. This exceedance should be interpreted as a conservative screening indicator associated with the assumptions of the deterministic US EPA methodology rather than evidence of actual adverse health effects. The one-at-a-time (OAT) sensitivity analysis confirmed that this finding was robust to moderate (±20%) variations in the principal exposure parameters. The total individual carcinogenic risk index was estimated at 2.0 × 10^−5^ for adults and 4.4 × 10^−5^ for children, which is within the acceptable risk range according to established classification standards.

The soils of southern Kazakhstan do not exhibit anomalous concentrations of natural radionuclides; the maximum specific activities recorded fall within the upper bounds of activity ranges reported for soils in other regions of the world. However, in several sampling locations, specific activity levels of Pb-210 exceed those of U-238, suggesting potential mechanisms for the enrichment of surface soils with Pb-210.

In all sampling points, the calculated Annual Effective Dose values remain below the ICRP recommended limits. The Excess Lifetime Cancer Risk (ELCR) ranges from 0.19 × 10^−3^ to 0.30 × 10^−3^, with an average value of 0.24 × 10^−3^. For most sampling sites, ELCR values do not exceed the UNSCEAR threshold value of 0.29 × 10^−3^, above which a slight cancer risk may be present. Only two locations show slight exceedances, with ELCR values reaching 0.30 × 10^−3^. Overall, it can be concluded that the radiological conditions in the study area are within normal limits. The influence of uranium mineralization and mining activities on the radiological environment in southern Kazakhstan is minimal.

The analysis of the spatial heterogeneity in the distribution of elements and radionuclides in the topsoil of the region reveals the following: 1. Trace contamination from the uranium deposit is observed in the surface soil layer, likely resulting from the accumulation of uranium decay products derived from the gaseous isotope Rn-222. This effect is manifested by elevated Pb-210 specific activity relative to U-238 in certain sampling points. 2. No evidence was found for the influence of potential pollutant transport by the Syr Darya River on the trace element or radionuclide composition of the regional soils. 3. No significant manifestations of anthropogenic impact were detected.

Thus, based on the obtained mean concentrations of chemical elements and radionuclides at different points of the upper soil layer of the study region, the spatial distribution of concentrations across the region, the comparison with the selected background site, as well as with the mean elemental concentrations in the upper continental crust and the ranges of radionuclide specific activities in Kazakhstan and other countries of the world, it can be concluded that the ore field of the uranium deposit and the technological operations associated with insitu leaching uranium mining have no fundamental influence on the elemental and radionuclide composition of the soils of the region.

## Figures and Tables

**Table 1:** Verification of Measurement Accuracy Using Certified Reference Materials.

Reference Material (Soil)	Radionuclide	CertifiedValue, Bq/kg	Relative Uncertainty of Certified Value, %	Decay-Corrected Certified Value, Bq/kg	MeasuredValue, Bq/kg
IAEA-375	Am-241 (I)	0.13	15	0.13 ± 0.02	<1.1
	Cs-137 (R)	5280	1.5	2460 ± 40	2540 ± 80
	K-40 (R)	424	1.7	424 ± 7	395 ± 25
	U-238 (I)	24.4	2.2	24.4 ± 5	21.5 ± 0.4
	Th-232(R)	20.5	1.8	20.5 ± 1.1	17.5 ± 0.5
IAEA-444	Am-241	55.6	2.9	55.6 ± 1.6	54.9 ± 1.3
	Cs-137	68.5	2.0	45.8 ± 0.9	46.3 ± 1.6
IAEA-Soil-6	Cs-137	53.65	4.1	20.0 ±0.6	22.2 ± 1.6
IAEA-RGU-1	U-238	4940	0.6	4940 ± 30	4830 ± 90
IAEA-RGTh-1	Th-232	3250	2.8	3250 ± 90	3150 ± 80

R—recommended activity value of the radionuclide; I—information value of radionuclide activity.

**Table 2:** Classification of Soil Contamination Based on Geoaccumulation Index (I_geo_).

Class	I_geo_ Value	Soil Quality Description
0	I_geo_≤0	practically uncontaminated
1	0 ≤ I_geo_< 1	uncontaminated to moderately contaminated
2	1 ≤ I_geo_< 2	moderately contaminated
3	2 ≤ I_geo_< 3	moderately to heavily contaminated
4	3 ≤ I_geo_< 4	heavily contaminated
5	4 ≤ I_geo_< 5	heavily to extremely contaminated
6	5 ≤ I_geo_	extremely contaminated

Class 6 is an open-ended class and includes all geoaccumulation index (I_geo_) values exceeding those of Class 5.

**Table 3:** Key Parameters of the Risk Assessment Model.

Parameter	Abbrev.	Unit	Children	Adults	Source
HMs concentration in soil	Ci	mg/kg	-	-	-
Ingestion rate	IngR	mg/day	200	100	[48,49,50]
Exposurefrequency	EF	days/year	350	350	[48]
Exposure duration	ED	years	6	24	[48]
Body weight	BW	kg	15	70	[47,48]
Average non-carcinogenic time	ATnc	days	2100	8400	[47]
Average carcinogenic time	ATca	days	25,550	25,550	[47]
Conversion factor	CF	kg/mg	10^−6^	10^−6^	[48,50]
Skin surface area	SA	cm^2^	2800	5700	[48]
Adherence factor	AF	mg/(cm^2^·day)	0.2	0.07	[48]
Dermal absorption factor	ABS	-	0.001	0.001	[48]
Inhalation rate	InhR	m^3^/day	7.6	20	[48,50]
Particulate emission factor	PEF	m^3^/kg	1.36·10^9^	1.36·10^9^	[48,50]
Lifetime	LT	years	70	70	[47]
Reference dose	RfD	mg/(kg·day)	Table 4		-
Cancer slope factor	SF	kg·day/mg	Table 4		-

**Table 4:** The reference doses (RfD) and cancer slope factors (SF) for HMs.

HMs	RfDing	RfDdermal	RfDinh	SFing	SFdermal	SFinh	Source
	mg/(kg·day)			kg·day/mg			
V	0.007	0.00007	0.007				[48]
Cr	0.003	0.00006	0.0000286	0.5	20	42	[48,50,51]
Mn	0.14	0.00184					[48]
Fe	0.7						[48,49,50,51,52]
Co	0.02	0.016					[48]
Ni	0.02	0.0054	0.0206				[48,50]
Cu	0.04	0.012	0.0402				[48]
Zn	0.3	0.06	0.3				[48,50]
Mo	0.005						[48,50]
Pb	0.0035	0.000525	0.00352	0.0085		0.042	[48,51]
U	0.003						[48]
Sb	0.0004						[48]
Ba	0,2						[48,51]

**Table 5:** Summary Statistics of Trace Element Concentrations in Soil of the Studied Region, µg/g.

Element	Mean	Standard Deviation	Maximum	Minimum	Background Value in the Studied Region (Point 9)	Concentration in the Upper Continental Crust [66]
Ti	2130	415	3010	1020	2290 ± 230	3930
V	65	14	110	31	63 ± 8	120
Cr	72	15	116	50	85 ± 10	92.4
Mn	457	79	621	301	510 ± 60	770
Fe	22,300	3300	31,600	16,700	23,700 ± 1000	40,600
Co	5.9	1.4	9.7	2.7	6.5 ± 0.8	17
Ni	35	9	62	22	43 ± 5	50
Cu	28	6	43	15	35 ± 4	39
Zn	66	23	132	25	82 ± 9	75
Rb	84	7	107	67	84 ± 13	98
Sr	327	94	728	207	311 ± 22	270
Y	16	2	21	11	16 ± 3	26
Zr	130	22	222	89	119 ± 12	160
Nb	8.2	0.9	10.7	6.3	8.3 ± 1.8	12
Mo	2.4	0.8	4.3	1.1	3.1 ± 0.7	1.56
Pb	26	10	77	16	52 ± 8	17
Th	7.4	1.6	10.9	3.3	7.0 ± 1.5	9.1
U	3.5	0.5	5.4	2.6	3.0 ± 0.7	2.5
Sn	7.0	0.8	9.4	5.4	7.9 ± 1.8	3.5
Sb	3.8	0.7	5.8	1.5	4.6 ± 1.1	0.81
Ba	610	70	834	471	589 ± 41	510
La	21	3	28	15	22 ± 2	32
Ce	50	5	62	41	50 ± 5	63
Pr	9.4	1.1	12.4	6.8	8.7 ± 1.3	8.7
Nd	24	1	27	21	23 ± 3	29
Sm	4.4	1.1	7.1	2.2	5.3 ± 1.6	5.7

**Table 6:** Specific Activities of Gamma-Emitting Radionuclides in Soil of the Studied Region, Bq/kg.

Radionuclide	Mean	Standard Deviation	Minimum	Maximum
^137^Cs	1.17	1.39	0.19	6.74
^40^K	614	53	487	792
^232^Th	28.0	5.8	17.3	40.0
^238^U	28.9	9.1	16.1	61.3
^226^Ra	26.5	4.8	17.1	34.5
^210^Pb	31.3	10.3	12.8	64.4

**Table 7:** The comparison of natural radioactivity levels in soil samples of different countries, Bq/kg.

Region of Study	^232^Th	^226^Ra	^40^K	^210^Pb	^238^U
	Range (Mean)				
Hungary	12–45 (28)	44–76 (33)	79–570 (370)		12–66 (29)
Switzerland	4–70 (25)	10–900 (40)	40–1000 (370)		10–150 (40)
United States	4–130 (35)	8–160 (40)	100–700 (370)	(107.3)	4–140 (35)
Russian Federation	2–79 (30)	1–76 (27)	100–1400 (520)		0–67 (19)
China	1–360 (41)	2–440 (32)	9–1800 (440)		2–690 (33)
India	14–160 (64)	7–81 (29)	38–760 (400)	(85.5)	7–81 (29)
Syrian	10–32 (20)	13–32 (20)	87–780 (270)		10–64 (23)
Algeria	2–140 (25)	5–180 (50)	66–1150 (370)		2–110 (30)
Turkey				30.8–177.6	
Kazakhstan	10–220 (60)	12–120 (35)	100–1200 (300)		12–120 (37)
World Average	45	32	420		33
Southern Kazakhstan—this study	17.3–40.0 (28.0)	17.1–34.5 (26.5)	487–792 (614)	12.8–64.4 (31.3)	16.1–61.3 (28.9)

**Table 8:** Classification of Soil Contamination Factor by Twelve Elements.

Element	Contamination Factor, $C_{f}^{i}$	Contamination Level
Cr	0.80	low contamination
Mn	2.6	moderate contamination
Ni	0.38	low contamination
Cu	0.56	low contamination
Zn	0.38	low contamination
Pb	0.37	low contamination
La	0.70	low contamination
Ce	0.78	low contamination
Pr	1.3	moderate contamination
Nd	0.42	low contamination
Sm	0.99	low contamination
Y	0.71	low contamination

**Table 9:** Results of non-carcinogenic risk assessment for adult and child population groups.

Risk Indices	Adults	Children
HQ_ing_	$\frac{\left(0.11÷0.17\right)}{0.13}$	$\frac{\left(1.0÷1.5\right)}{1.2}$
HQ_dermal_	$\frac{\left(0.011÷0.018\right)}{0.014}$	$\frac{\left(0.07÷0.12\right)}{0.09}$
HQ_inh_	$\frac{\left(3.7\times 10^{-4}÷6.4\times 10^{-4}\right)}{5.3\times 10^{-4}}$	$\frac{\left(6.5\times 10^{-4}÷1.1\times 10^{-3}\right)}{9.4\times 10^{-4}}$
HI_average_	0.15	1.3

**Table 10:** Results of carcinogenic risk index calculation for adults and children.

Element	Adults			CR_adults_	Children			CR_children_
	CR_ing_	CR_dermal_	CR_inh_		CR_ing_	CR_dermal_	CR_inh_	
Cr	1.7 $\times 10^{-5}$	2.7 $\times 10^{-6}$	2.1 $\times 10^{-7}$	2.0 $\times 10^{-5}$	3.9 $\times 10^{-5}$	4.4$\times 10^{-6}$	9.2 $\times 10^{-8}$	4.4 $\times 10^{-5}$
Pb	1.0 $\times 10^{-7}$		7.2 $\times 10^{-11}$	1.0 $\times 10^{-7}$	2.3 $\times 10^{-7}$		3.2 $\times 10^{-11}$	2.3 $\times 10^{-7}$
CR_adults_				2.0 $\times 10^{-5}$	CR_children_			4.4 $\times 10^{-5}$

**Table 11:** Summary Statistics of Radiological Parameters.

Radiological Parameter	Mean	Standard Deviation	Minimum	Maximum
Annual Effective Dose (mSv/y)	0.067	0.008	0.053	0.087
Lifetime cancer risk × 10^−3^	0.24	0.03	0.19	0.30

**Table 12:** Comparison of radiological parameters with uranium mining areas worldwide.

Region/Country	Mining Type	Annual Effective Dose, mSv/y	ELCR ×10^−3^	Reference
Syr Darya province, Kazakhstan	Uranium, ISL	0.067 (0.053–0.087)	0.24 (0.19–0.30)	This study
Rössing, Namibia	Uranium, open-pit	0.30 (0.12–1.60)	—	[79]
Singhbhum, India	Uranium, underground	0.11–0.14	0.27	[80]
South China	Uranium, open-pit + tailings	2–10 times the world average	elevated	[81]
World average (outdoor)	—	0.07	0.29	[78]

**Table 13:** Pearson Correlation Matrix of Chemical Elements.

Variable	Correlations (Elements) Marked Correlations Are Significant at *p* < 0.05000 N = 30 (Casewise Deletion of Missing Data)																									
	Ti	V	Cr	Mn	Fe	Co	Ni	Cu	Zn	Rb	Sr	Y	Zr	Nb	Mo	Pb	Th	U	Sn	Sb	Ba	La	Ce	Pr	Nd	Sm
Ti	1																									
V	0.22	1																								
Cr	−0.08	0.15	1																							
Mn	**0.89**	0.07	−0.04	1																						
Fe	**0.84**	0.11	−0.11	**0.89**	1																					
Co	**0.71**	−0.09	0.07	**0.86**	**0.87**	1																				
Ni	**0.49**	0.22	0.24	**0.48**	**0.68**	**0.58**	1																			
Cu	0.35	−0.27	0.04	**0.55**	**0.66**	**0.72**	**0.67**	1																		
Zn	**0.55**	−0.16	−0.18	**0.69**	**0.78**	**0.69**	**0.54**	**0.78**	1																	
Rb	0.17	0.11	0.05	0.11	0.43	0.34	**0.62**	0.32	0.21	1																
Sr	0.09	−0.07	0.10	0.28	0.21	0.21	0.21	**0.46**	**0.44**	−0.33	1															
Y	**0.87**	0.36	0.05	**0.82**	**0.79**	**0.67**	**0.43**	0.21	0.34	0.21	−0.07	1														
Zr	**0.62**	0.30	0.01	**0.50**	0.27	0.25	−0.06	−0.19	−0.08	−0.26	−0.12	**0.70**	1													
Nb	**0.87**	0.26	−0.01	**0.79**	**0.69**	**0.58**	0.31	0.14	0.28	0.16	−0.17	**0.88**	**0.69**	1												
Mo	0.30	−0.12	−0.19	**0.48**	**0.51**	**0.48**	0.32	**0.53**	**0.59**	−0.04	**0.49**	0.22	−0.02	0.02	1											
Pb	**0.41**	−0.12	0.09	**0.55**	**0.55**	**0.51**	**0.43**	**0.63**	**0.60**	0.19	0.20	0.32	−0.05	0.27	**0.47**	1										
Th	**0.71**	0.27	−0.06	**0.67**	**0.68**	**0.61**	0.30	0.14	0.21	0.32	−0.18	**0.85**	**0.60**	**0.80**	0.14	0.22	1									
U	−0.01	0.05	0.21	0.09	0.17	0.20	0.25	0.29	0.15	0.00	**0.67**	0.04	−0.17	−0.16	0.18	−0.08	−0.05	1								
Sn	0.22	0.15	−0.13	0.25	0.20	0.28	−0.06	0.02	0.05	0.07	−0.25	0.26	0.29	0.31	0.10	0.26	0.25	−0.13	1							
Sb	0.27	−0.31	−0.07	**0.44**	**0.39**	**0.64**	0.07	**0.42**	**0.42**	0.10	−0.23	0.25	0.06	0.28	0.20	**0.37**	0.24	−0.24	**0.45**	1						
Ba	**−0.69**	−0.14	0.06	**−0.79**	**−0.62**	**−0.53**	−0.23	**−0.39**	**−0.50**	0.33	**−0.53**	**−0.64**	**−0.56**	**−0.63**	**−0.44**	−0.36	**−0.49**	−0.31	−0.21	−0.12	1					
La	**0.93**	0.31	−0.06	**0.85**	**0.82**	**0.71**	**0.50**	0.34	**0.42**	0.22	0.00	**0.92**	**0.65**	**0.91**	0.24	**0.37**	**0.76**	0.05	**0.38**	0.30	**−0.70**	1				
Ce	**0.92**	0.28	−0.06	**0.84**	**0.82**	**0.72**	**0.49**	0.34	**0.42**	0.21	−0.03	**0.93**	**0.66**	**0.89**	0.24	0.30	**0.77**	0.04	0.34	0.30	**−0.68**	**0.98**	1			
Pr	0.12	−0.06	−0.12	0.10	0.16	0.22	0.01	0.00	−0.06	0.29	**−0.37**	0.17	0.00	0.28	−0.20	−0.10	**0.43**	−0.19	0.07	0.27	0.14	0.18	0.22	1		
Nd	**0.69**	0.27	−0.01	**0.50**	**0.58**	**0.51**	**0.39**	0.07	0.11	**0.43**	−0.34	**0.74**	**0.54**	**0.70**	−0.04	0.04	**0.72**	−0.18	0.13	0.20	−0.19	**0.71**	**0.76**	**0.37**	1	
Sm	0.20	0.20	0.12	0.16	0.07	0.16	−0.06	−0.17	−0.11	0.12	−0.34	0.33	**0.41**	0.26	−0.05	0.21	**0.49**	−0.29	0.22	0.27	0.03	0.24	0.18	0.24	0.31	1

**Table 14:** Pearson Correlation Matrix of Gamma-Emitting Radionuclides.

	^137^Cs	^40^K	^232^Th	^238^U	^226^Ra	^210^Pb
^137^Cs	0.00					
^40^K	0.75	0.00				
^232^Th	0.64	1.12	0.00			
^238^U	0.67	1.26	0.39	0.00		
^226^Ra	0.72	1.17	0.16	0.48	0.00	
^210^Pb	0.74	1.23	0.46	0.41	0.49	0.00

## Data Availability

The original contributions presented in this study are included in the article/Supplementary Materials. Further inquiries can be directed to the corresponding author.

## Supplementary Materials

The following supporting information can be downloaded at https://www.mdpi.com/article/10.3390/toxics14080665/s1. Table S1: Soil Sampling Locations; Table S2: Limits of Detection (LOD) of the X-ray Fluorescence Method in Soil Sample Analysis; Table S3: One-at-a-time (OAT) sensitivity analysis of deterministic human health risk assessment; Table S4: Eigen values; Table S5: Factor loadings (|r| ≥ 0.5), PC1–PC2; Figure S1: Loading Plot.
