# Supplementary material for: Assessment of the Environmental Impact of Uranium Mining Sites: A Case Study of a Uranium Deposit in Southern Kazakhstan
Source: Toxics. 2026 Jul 27;14(8):665. doi: 10.3390/toxics14080665 (PMC13517581; doi:10.3390/toxics14080665)
Supplement: Supplementary file 1 [file toxics-14-00665-s001.zip › toxics-4424426-supplementary.pdf]

Table 1 Soil Sampling Locations

№	Site	Study Location	Latitude (N)	Longitude (E)	Number of Soil Samples	Justification for Sampling Site Selection
1	1-NP	Baikenzhe village	43° 53′ 14.14″	66° 55′ 10.11″	3	Proximity to the Kharasan uranium mine
2	2-NP	Bilibaı village	43° 53′ 43.13″	66° 59′ 05.46″	3	
3	3-NP	Akmaya village	44° 05′ 08.85″	66° 51′ 53.27″	3	Proximity to the Northern Karamurun mine
4	4-NP	Bidaikol village	44° 08′ 15.86″	66° 44′ 22.84″	3	
5	5-NP	Baisyn village	44° 08′ 35.32″	66° 42′ 01.07″	3	Location within the potential impact zone, near the canal network
6	6-NP	Sh. Kodamanov village	44° 10′ 02.96″	66° 49′ 07.81″	3	Located along the transportation route of low-level radioactive waste to the disposal site. Shieli is a major settlement within the potential impact zone
7	7-NP	Shieli village	44° 10′ 16.38″	66° 43′ 40.38″	5	
8	8-NP	Y. Zhakaev village	44° 10′ 16.38″	66° 41′ 36.49″	3	Located within the potential impact zone, near the canal network
9	9-NP	Bulanbaibauy village	43° 54′ 00.89″	66° 36′ 57.13″	3	Located outside the potential impact zone; can serve as a control site for comparison with other villages
10	10-NP	N. Bekezhanov village	44° 11′ 36.83″	66° 46′ 46.64″	3	Located along the transportation route of low-level radioactive waste to the disposal site
11	11-NP	Enbekshi village	44° 14′ 19.47″	66° 50′ 21.03″	3	
12	12-NP	Aktam village	43° 59′ 12.74″	66° 43′ 08.90″	2	Located within the potential impact zone, near a system of canals fed by the Syr Darya River
13	13-ZM	winter camp	43° 53′ 32.91″	66° 47′ 32.91″	2	Located near the Kharasan mine
14	14-Zm	winter camp, Kharasan mausoleum	43° 45′ 33.12″	66° 54′ 35.43″	2	Located south of the Kharasan mining site, in proximity to a population gathering point (mausoleum)
15	15-ZM	winter camp	44° 02′ 31.25″	66° 51′ 11.82″	2	Located in the vicinity of the Yuzhny Karamurun mine
16	16-ZM	winter camp	43° 59′ 09.25″	66° 51′ 41.02″	2	
17	17-ZM	winter camp	44° 00′ 59.05″	66° 49′ 19.31″	2	
18	18-ZM	winter camp	44° 02′ 47.85″	66° 47′ 57.21″	2	
19	19-ZM	winter camp	43° 52′ 46.23″	66° 46′ 03.12″	2	Located near the Kharasan mine
21	21-K	Smallholder farm	43° 52′ 11.64″	66° 55′ 07.03″	1	
22	22-KH	Smallholder farm	43° 48′ 40.97″	66° 57′ 54.90″	2	
23	23-ZM	winter camp	44° 06′ 55.27″	66° 31′ 55.79″	2	Located in the vicinity and within the mining lease area

№	Site	Study Location	Latitude (N)	Longitude (E)	Number of Soil Samples	Justification for Sampling Site Selection
24	24-ZM	winter camp	44° 07′ 24.58″	66° 34′ 12.73″	2	of the Irkol uranium mine.
25	25-ZM	winter camp	44° 04′ 21.52″	66° 30′ 58.30″	2	
26	26-P	pontoon	43° 56′ 56.01″	66° 42′ 36.42″	1	Downstream of the Syrdarya River, beyond the mining area
27	27-P	Syrdarya River	43° 58′ 07.84″	66° 43′ 46.21″	1	
28	28-P	Tomenaryk cross-section	44° 00′ 51.70″	67° 01′ 16.88″	1	Upstream of the Syrdarya River, before the mining area
29	29-K	Kharasan canal	43° 54′ 50.35″	66° 51′ 12.76″	1	A canal crossing the mining lease area of the Kharasan deposit
30	30-K	Irrigation canal	44° 07′ 36.64″	66° 46′ 32.89″	1	Located in the vicinity of the Severny Karamurun deposit
31	31-K	Discharge canal	44° 06′ 09.44″	66° 46′ 49.36″	1	
				**Total**	**66**	

Table S2. Limits of Detection (LOD) of the X-ray Fluorescence Method in Soil Sample Analysis

Element	Excitation Source	Detection Limit, µg/g	Element	Excitation Source	Detection Limit, µg/g
**K**	^{109}Cd	3000	**Zr**	^{109}Cd	1.2
**Ca**	^{109}Cd	2000	**Nb**	^{109}Cd	1.2
**Ti**	^{109}Cd	300	**Mo**	^{109}Cd	1.6
**V**	^{109}Cd	150	**Hf**	^{109}Cd	37
**Cr**	^{109}Cd	70	**W**	^{109}Cd	30
**Mn**	^{109}Cd	50	**Pb**	^{109}Cd	7.5
**Fe**	^{109}Cd	40	**Bi**	^{109}Cd	6.0
**Co**	^{109}Cd	25	**Th**	^{109}Cd	3.3
**Ni**	^{109}Cd	15	**U**	^{109}Cd	2.5
**Cu**	^{109}Cd	10	**Ag**	^{241}Am	1.8
**Zn**	^{109}Cd	9.0	**Cd**	^{241}Am	1.6
**Ga**	^{109}Cd	5.0	**Sb**	^{241}Am	1.5
**Rb**	^{109}Cd	1.5	**Cs**	^{241}Am	1.3
**Ge**	^{109}Cd	5.0	**Ba**	^{241}Am	1.2
**As**	^{109}Cd	20	**La**	^{241}Am	1.1
**Se**	^{109}Cd	3.0	**Ce**	^{241}Am	1.2
**Br**	^{109}Cd	2.5	**Pr**	^{241}Am	1.3
**Sr**	^{109}Cd	1.4	**Nd**	^{241}Am	1.5
**Y**	^{109}Cd	1.3	**Sm**	^{241}Am	2.6

The analytical results of the measured concentrations of chemical elements were presented in the form: C ± Δ, where C is the concentration of the determined element (µg/g), and Δ is the calculated uncertainty of the measured concentration (µg/kg).

Table S3 - One-at-a-time (OAT) sensitivity analysis of deterministic human health risk assessment

Parameter	Variation	HQing	HQderm	HQinh	HI	CR	Change relative to initial data in HI	Change relative to initial data in CR
				Adult				
Initial data	–	0.13	0.014	5.3×10-4	0.15	2.0×10-5	–	–
Ci	-20%	0.11	0.011	4.2×10-4	0.12	1.6×10-5	-20%	-20%
	20%	0.16	0.017	6.3×10-4	0.18	2.4×10-5	20%	20%
IngR	-20%	0.11	0.014	5.3×10-4	0.12	1.6×10-5	-18%	-17%
	20%	0.16	0.014	5.3×10-4	0.17	2.3×10-5	18%	17%
BW	-20%	0.16	0.018	6.6×10-4	0.18	2.5×10-5	25%	25%
	20%	0.11	0.012	4.4×10-4	0.12	1.6×10-5	-17%	-17%
ED	-20%	0.13	0.014	5.3×10-4	0.15	1.6×10-5	0%	-20%
	20%	0.13	0.014	5.3×10-4	0.15	2.4×10-5	0%	20%
				Child				
Initial data	–	1.23	0.092	9.4×10-4	1.32	4.4×10-5	–	–
Ci	-20%	0.99	0.074	7.5×10-4	1.06	3.5×10-5	-20%	-20%
	20%	1.48	0.111	1.1×10-4	1.59	5.3×10-5	20%	20%
IngR	-20%	0.99	0.092	9.4×10-4	1.08	3.6×10-5	-19%	-18%
	20%	1.48	0.092	9.4×10-4	1.57	5.2×10-5	19%	18%
BW	-20%	1.54	0.115	1.2×10-4	1.66	5.5×10-5	25%	25%
	20%	1.03	0.077	7.8×10-4	1.10	3.7×10-5	-17%	-17%
ED	-20%	1.23	0.092	9.4×10-4	1.32	3.5×10-5	0%	-20%
	20%	1.23	0.092	9.4×10-4	1.32	5.3×10-5	0%	20%

S4. Principal Component Analysis

To provide a source-oriented interpretation complementing the correlation and cluster analyses, principal component analysis (PCA) was performed on the concentrations of 26 elements (Ti, V, Cr, Mn, Fe, Co, Ni, Cu, Zn, Rb, Sr, Y, Zr, Nb, Mo, Pb, Th, U, Sn, Sb, Ba, La, Ce, Pr, Nd, Sm) in 30 samples. The analysis was carried out in STATISTICA 10 on standardized data using the correlation matrix. Components were retained following the Kaiser criterion (eigenvalue > 1), and loadings with $|r| \geq 0.50$ were considered significant for interpretation. Five components satisfied the Kaiser criterion and together explained 79.1% of the total variance (Table S4). The interpretation is based on the first two components (56.5% of the variance); the higher-order components (PC3–PC5, 22.6% combined) reflect minor local variability of individual elements and were not interpreted in terms of sources.

The first component (PC1, 40.5%) shows high, same-sign loadings for the rare-earth and high-field-strength elements (La, Ce, Y, Nb, Th, Nd), the Ti–Fe–Mn–Co group, and Ni, Cu, Zn and Pb (Table S5, Figure S1), and is interpreted as a lithogenic factor reflecting the composition of the parent material and weathering products. Barium loads with the opposite sign, indicating geochemically independent behaviour. The second component (PC2, 16.0%) is defined by Sr, Cu, Zn, Mo and U and is interpreted as a natural geochemical-mobility factor separating the more mobile elements from the rare-earth/Zr association, consistent with the U–Sr association identified in the correlation analysis. Chromium loads near zero on both PC1 and PC2, indicating that its variability is not controlled by these factors. The PCA is consistent with the dendrogram, in which La–Ce–Ti–Y–Nb, Sr–U and Fe–Mn–Co form distinct groupings, confirming the stability of the solution and the predominantly natural origin of the elemental associations.

Table S4 — Eigen values:

Component	Eigen value	% of variance	Cumulative %
PC1	10.53	40.5	40.5
PC2	4.15	16.0	56.5
PC3	2.55	9.8	66.3
PC4	2.03	7.8	74.1
PC5	1.30	5.0	79.1

Table S5 — Factor loadings (|r| ≥ 0.5), PC1–PC2:

Element	PC1	PC2
La	−0.94	
Mn	−0.94	
Ce	−0.94	
Fe	−0.93	
Ti	−0.93	
Y	−0.91	
Co	−0.86	
Nb	−0.85	
Th	−0.79	
Nd	−0.68	
Zn	−0.62	+0.63
Ni	−0.58	
Zr	−0.54	−0.54
Cu	−0.53	+0.71
Pb	−0.51	
Sr		+0.77
Mo		+0.59
U		+0.51
Ba	+0.71	

Sign of the loadings is arbitrary. Cr is omitted (|loading| < 0.5 on both components).

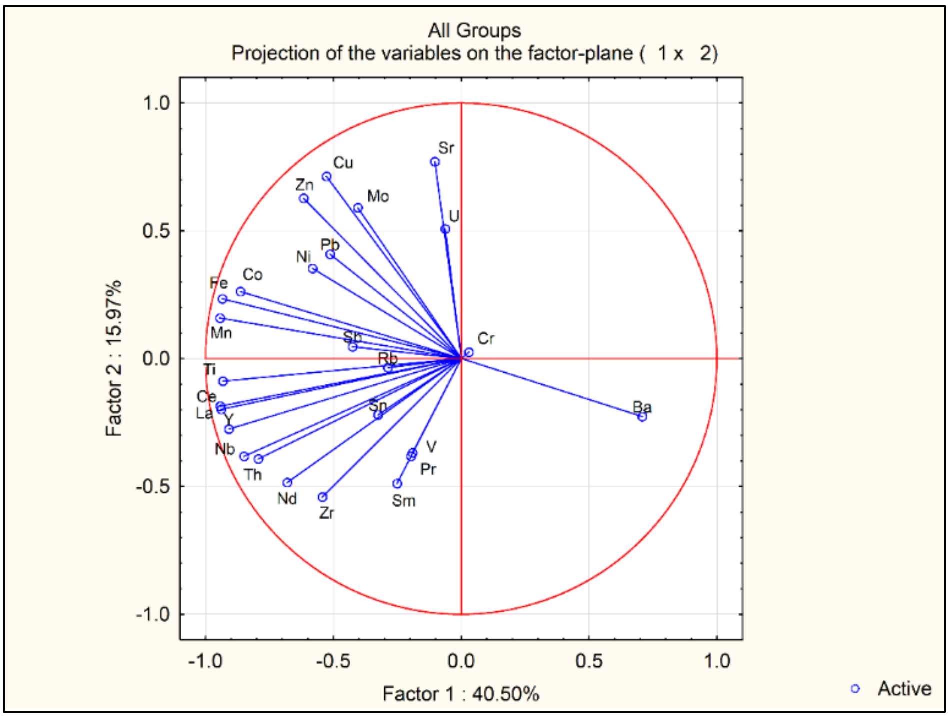

Figure S1. Loading Plot
